# Modulation of mitochondrial voltage dependent anion channel: studies on bilayer electrophysiology

**DOI:** 10.3389/fphys.2025.1666994

**Published:** 2025-12-03

**Authors:** Daniel Tuikhang Koren, Chetan Malik, Shumaila Iqbal Siddiqui, Rajan Shrivastava, Subhendu Ghosh

**Affiliations:** 1 Department of Physiology & Biophysics, School of Medicine, University of California, Irvine, Irvine, CA, United States; 2 Department of Biophysics, University of Delhi-South Campus, New Delhi, India; 3 Virginia-Maryland College of Veterinary Medicine, Virginia Tech, Blacksburg, VA, United States; 4 School of Computer Science and Engineering, Vellore Institute of Technology, Vellore, Tamil Nadu, India

**Keywords:** voltage-dependent anion channel (VDAC), bilayer electrophysiology, mitochondrial dysfunction, post-translational modifications, ligand interactions, protein phosphorylation, oxidative stress, apoptosis

## Abstract

The present paper is a review of the mitochondrial Voltage Dependent Anion Channel (VDAC), popularly known as mitochondrial porin, which is a protein that forms a passive diffusion ion channel across the outer membrane of the mitochondrion. VDAC essentially plays an important role in the transport of metabolites like ATP between the intermembrane space of the mitochondrion and the cytoplasm. However, under certain conditions, it can give rise to cellular dysfunction, e.g., apoptosis. Although VDAC is present in all eukaryotic cells, this review has focused mainly on the animal tissues. Interactions of VDAC with various enzymes, proteins, and small molecules or ligands have been reviewed with a perspective of bilayer electrophysiology. Importantly, the biochemical (post-translational) modifications of the channel protein, namely, phosphorylation (by a series of kinases), acetylation, ubiquitination, oxidative modifications (such as glutathionylation and nitrosylation), etc., and their impact on the electrophysiological properties have been discussed. Finally, the consequences of the above-mentioned experimental findings have been discussed with predictions and hypotheses relevant to living systems.

## 1 Introduction

Mitochondrial porin, or voltage-dependent anion channel (VDAC), has been identified to control the permeability of the outer membrane of the mitochondrion (Schein et al., 1976; De Pinto et al., 1985). In animal tissues, VDAC is known to have three isoforms (Messina et al., 2012) with molecular masses ranging from 30 to 32 kDa (Blachly-Dyson et al., 1993; Sampson et al., 1997; Hinsch et al., 2004; Messina et al., 2012). However, in plants and other tissues, VDAC has been reported to exist in some more isoforms with different molecular weights (Elkeles et al., 1997; Homblé et al., 2012; Salinas et al., 2014; Saidani et al., 2017; Rodríguez-Saavedra et al., 2023). More details are given in a separate section of this review. VDAC essentially plays an important role in the transport of metabolites like ATP between the intermembrane space (IMS) of the mitochondrion and the cytoplasm. It forms a large voltage-gated pore (2.5–3 nm diameter) and acts as the pathway for the movement of substances in and out of the mitochondria by passive diffusion (De Pinto et al., 1985). Whether isolated from humans or any other organisms, VDAC shows a remarkably conserved set of biochemical and biophysical properties (Magrì et al., 2019; Di Rosa et al., 2021). When reconstituted in bilayer lipid membrane (BLM), all VDACs show roughly similar single-channel conductance (4.1–4.5 nS in 1M KCl) and cation preference in closed or lower conductance states (Benz, 1988; Ludwig et al., 1988; Benz et al., 1990; Smart et al., 1997; Colombini, 2004). Interestingly, it has been demonstrated that VDAC contributes to the electrical capacitance of the lipid bilayer membrane (Ghosh et al., 1999; Ghosh and Bera, 2001).

The importance of mitochondrial VDAC increased when it was found to play a crucial role in apoptotic cell death (Shimizu et al., 1999; 2000; Madesh and Hajnóczky, 2001). Apoptosis is known to take place in cells through two major pathways: the death receptor and the mitochondrial pathways (Jonas et al., 1999). Under an abnormal condition, the release of pro-apoptotic substances, such as the apoptosis-inducing factor (Susin et al., 1999), endonuclease G (Li et al., 2001; Zhang et al., 2014), Smac/DIABLO (Du et al., 2000; Verhagen et al., 2000), and cytochrome c (Adams and Cory, 2001), is responsible for the mitochondria-mediated cell death via activation of apoptotic biochemical networks, e.g., the cytochrome c-mediated pathway (Jiang and Wang, 2004; Clayton et al., 2005). Cytochrome c is released into cytosol through VDAC in the presence of the B-cell lymphoma 2 (Bcl 2) family proteins, Bcl-2 Associated X (Bax) protein (apoptosis regulator) and truncated BH3 Interacting Domain Death agonist (tBid), during apoptosis as a result of an increase in the pore size of VDAC (Shimizu et al., 1999; Tsujimoto and Shimizu, 2000; Banerjee and Ghosh, 2004a). Release of cytochrome c from mitochondria inactivates the electron transport chain and causes cell death (Krippner et al., 1996). On the other hand, closure of the VDAC pore leads to the inhibition of metabolite (ATP) transport from the mitochondria to the cytosol. And the obvious consequence is disruption of biochemical function and swelling and bursting of mitochondria, hence cell death. How do living cells resist the aforesaid processes? When do the cells fail to offer this resistance and succumb to death? Understanding the modulation of VDAC would play an important role in answering all these questions.

As of now, there is a plethora of experimental evidence displaying various modes of modulation of VDAC. As many, if not all, ion channels are subject to post-translational modifications by phosphorylation, sulphonation, acetylation, etc., which play a particularly important role in modulating VDAC (Levitan, 1988; Levitan, 1994). Similarly, several different protein kinases can participate in the regulation of VDAC properties, and it is not unusual to discover that VDAC is modulated by several different protein kinases, each influencing the channel activity in a unique way.

In this paper, we have reviewed the reports on the role of biochemical modifications, e.g., phosphorylation, sulphonation, acetylation, etc., in the modulation of VDAC and its interaction with various proteins and small molecules or ligands. The investigations are based on bilayer electrophysiological (BLM) experiments. In this review, the following kinases and their effects have been given a major emphasis: Protein Kinase A & C (PKA/C), c-Jun N-terminal Kinase 3 (JNK3), Extracellular Signal-Regulated Kinase (ERK), and Calmodulin Kinase II (CaMK-II). And the proteins interacting with VDAC, mainly considered and discussed in this review, are as follows: plasminogen, Bax, Bid, Bif, CaM, hexokinase, etc. Moreover, some small molecules or ligands interacting with VDAC that are being discussed here include homocysteine, HgCl_2_, H_2_O_2_, N-acetyl L-cysteine (NAC), quinidine, and many more. In addition, we have discussed some biophysical studies, e.g., clustering, noise & fractal analyses of VDAC and their relevance in understanding the functioning of this ion channel.

### 1.1 VDAC sources, location, and isoforms

VDAC proteins are found in several organisms, which include animals, plants, and fungi (Homblé et al., 2012). Three VDAC isoforms, such as VDAC 1, 2, and 3, are widely found in mammals, including humans, mice, and rats, and are reported to be expressed mainly in the outer mitochondrial membranes (Messina et al., 2012). VDAC1 is the most abundantly expressed in human tissues, VDAC2 is highly expressed in the reproductive system and nervous tissue, and VDAC3 is abundant in the testis (Zinghirino et al., 2021). mVDAC1, mVDAC2, and mVDAC3 share similar structures and expression patterns with humans (Zinghirino et al., 2021). Plants are reported to have variable isoforms from species to species (Homblé et al., 2012). Some fish species, such as zebrafish, express all three VDAC isoforms (Zhang et al., 2024). VDAC1 isoform was identified and well characterized in *Caenorhabditis elegans* (Ren et al., 2023) and *Drosophila melanogaster* (Blachly-Dyson et al., 1993). Moreover, some fungal species, such as *Saccharomyces cerevisiae*, have two isoforms (Di Rosa et al., 2021), while *Neurospora crassa* VDAC exists in a single isoform, but the core VDAC protein is highly conserved with significant structural and functional similarity to human VDAC1 (Freitag et al., 1982). Notably, VDAC proteins have been found in protists like *Paramecium aurelia* (Schein et al., 1976). In fact, VDAC was discovered and characterized for the first time in the mitochondria of *Paramecium aurelia* (Schein et al., 1976).

After decades of research, it is now established that the most abundant and well-characterized location for VDACs is the outer mitochondrial membrane (Schein et al., 1976; De Pinto et al., 1985). However, evidence had sprung up for their presence in other cellular compartments (Massa et al., 2000; Naghdi and Hajnóczky, 2016). Interestingly, VDAC has been reported to exist in the plasma membrane of animal cells (Elinder et al., 2005; De Pinto et al., 2010), though their roles and functions are still debated and require further investigations. Plasmalemmal VDAC (pl-VDAC) is the term used to designate these VDACs that are found in the plasma membrane. It has been established that pl-VDAC is present in red blood cells (RBCs), although it is not isoform-specific (Shimizu et al., 2001). VDAC2 protein was found in the sperm cells, specifically on the tail of the *Drosophila* spermatozoa (Guarino et al., 2006). Also, VDAC 2 & 3 were found in the flagellum’s outer dense fibers of bovine (Hinsch et al., 2004).

In humans, the acrosomal plasma membrane was found to contain VDAC2 (Liu et al., 2011), while in porcine oocytes, pl-VDAC1/2 expression was detected, but not VDAC3 (Carolina Cassar et al., 2010). Moreover, scientific data are poorly documented in the literature on the structural and functional role of VDAC2 in the plasma membrane. Some reports have revealed that the amount of iron was upregulated in the plasma membrane of K562 cells (erythroleukemia) when their cellular iron content was low (Valis et al., 2008). This report suggested that VDAC2 might have assisted the cells in uptaking iron (Valis et al., 2008). Likewise, some reports suggest that VDAC is involved and plays a significant role in the regulation of synaptic plasticity (Levy et al., 2003; Rosencrans et al., 2025). However, it is a debatable issue and demands rigorous investigation.

Early research proposed that VDAC was a structural part of the mPTP complex, along with the Adenine Nucleotide Translocase (ANT) in the inner mitochondrial membrane (IMM) and cyclophilin D (CypD) in the mitochondrial matrix (Szabó et al., 1993; Rao et al., 2014). However, key studies disproved this direct role, including a genetic knockout study (Craigen and Graham, 2008). Experiments with mice genetically engineered to lack all VDAC isoforms (VDAC1^−/−^, VDAC2^−/−^, and VDAC3^−/−^) showed that mitochondria from these mice could still undergo a permeability transition (Craigen and Graham, 2008). The current understanding is that VDAC plays a direct role in apoptosis by releasing pro-apoptotic proteins, such as cytochrome c, from the mitochondria. This mechanism is distinct from mPTP-induced cytochrome c release, which is caused by the osmotic swelling of the mitochondria following IMM permeabilization (Baines et al., 2003; Baines, 2009).

### 1.2 VDAC structure

**FIGURE 1:**
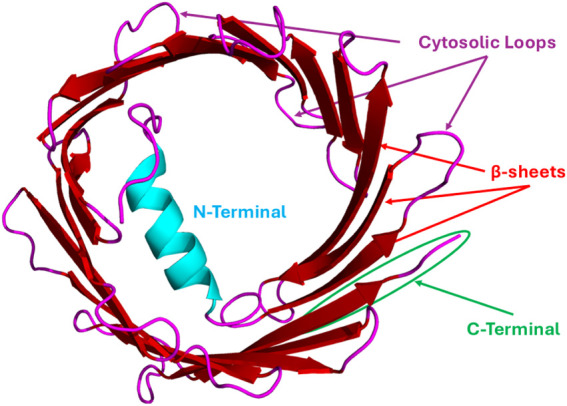
Representative VDAC Structure (human VDAC1): hVDAC1 generally consists of ∼283 amino acid residues and 19 β-strands/sheets with ∼25 residues in N-terminal flexible regions, and each strand is flanked by a loop on both the cytosolic and intermembrane space (IMS) sides. This structure was generated from the human VDAC1 sequence (UniProt ID: P21796) retrieved from the UniProt database and was subjected to the AlphaFold3 web server for structural prediction. The obtained structure was visualized by PyMOL software.

The 3D structures of the mouse and human VDAC1 isoform have been established through the use of biophysical methods like NMR spectroscopy and X-ray crystallography (Bayrhuber et al., 2008; Hiller et al., 2008; Ujwal et al., 2008). According to the structural analysis, 19 β-strands are arranged in a β-barrel along the membrane, and there is also a region with an α-helix at the N-terminus (see Figure 1). Strands 1 and 19 run in parallel, but otherwise the barrel is arranged as a regular antiparallel array of β-strands, and one can find the amphipathic α-helix tail inside the pore (Hiller et al., 2008). However, these characteristics do not exactly match the X-ray and NMR structures, and hence the precise location and local structure of this N-terminal α-helix remain elusive (Bayrhuber et al., 2008). A high-resolution structure of zebrafish VDAC2 confirms its β-barrel organization, like that of VDAC1 (Schredelseker et al., 2014). Zebrafish VDAC2 contains one cysteine residue and lacks the 11-amino-acid longer N-terminal sequence found in mammalian VDACs. VDAC3’s structure is yet to be determined. Several bioinformatic predictions, based on the substantial sequence similarity, postulated a barrel core like the other VDAC isoforms (Amodeo et al., 2014). Despite their significant sequence similarity and structural homology, VDAC isoforms have distinct functional properties within the cell.

Several distinct regions and specific amino acid residues of the VDAC protein are critical for its interactions with binding partners, enabling functions from metabolism to apoptosis. The specific residues involved vary depending on the VDAC isoform and the interacting protein (see Table 1). The flexible N-terminal region of VDAC, located inside the β-barrel pore (see Figure 1), is a major hub for protein interactions. This domain is crucial for binding to some key proteins like HK-I and the Bcl-2 family of proteins (Adams and Cory, 2001). Flexible and cytosol-facing loops are interspersed within the 19 β-strands that form the VDAC barrel structure. These loops are important for binding a variety of proteins. β-strands 7–10 cytosolic loop is a crucial interface for VDAC2’s interaction with the pro-apoptotic proteins like Bax and Bak (Banerjee and Ghosh, 2004a; Chin et al., 2018). The Ala171 residue, located within the β10–11 loop, is particularly important for VDAC2’s interaction with Bak (Chin et al., 2018). Mutations at this site can stabilize or destabilize the VDAC2-Bak complex and regulate apoptotic signaling. VDAC2 has nine cysteine residues (compared to only two in VDAC1). Some of these, located in the loops between β-strands, may be involved in redox regulation and protein–protein interactions (Catterall, 1988). During VDAC1 oligomerization, β-strands 1, 2, 16, and 19 form contact sites between individual VDAC1 monomers (Geula et al., 2012). These interaction sites involve residues exposed to the lipid bilayer (Geula et al., 2012). In VDAC1, Cys127 and Cys232 are highly unstable and can be cross-linked during oligomerization, indicating their involvement in forming higher-order VDAC structures (Geula et al., 2012). The C-terminal domain of VDAC also contains interaction sites. For instance, the cytoskeletal protein gelsolin binds to the C-terminus of VDAC1 to regulate channel activity (Kusano et al., 2000). Less or nothing is known to date about the structural information of other isoforms and those VDACs that originate from other organelles, as well as from diverse species in general.

**TABLE 1:** Phosphorylation of VDAC.

Kinase	Phosphorylation effect on VDAC	Plausible functional consequence	Known phosphorylation sites	References
Protein Kinase A (PKA)	Phosphorylates VDAC, reduces single-channel current	Regulates permeability for mitochondrial respiratory substrates	Unknown	Bera et al. (1995), Bera and Ghosh (2001), Sheldon et al. (2011)
JNK3	Phosphorylates VDAC, interferes with channel functioning	Closure and reopening of the channel; massive reduction in opening probability	Unknown	Gupta and Ghosh (2015b), Gupta and Ghosh (2017)
ERK1/2	Phosphorylates VDAC, reduces single-channel current	Physically interact with mitochondrial proteins and affect the transport mechanism of VDAC	Thr33, Thr55, Ser35	Galli et al. (2009), Malik et al. (2022)
CaMKII	Phosphorylates VDAC, drastically reducing channel current	Holds channel in open state, loss of voltage sensitivity; controls mitochondrial mobility	Unknown	Nguyen et al. (2018), Koren et al. (2023)
Protein Kinase C (PKC)	Phosphorylates VDAC	Functional consequences have not yet been studied electrophysiologically	Unknown	Baines et al. (2003)
GSK-3β	Phosphorylates VDAC2	Enables HK-I binding to VDAC, promotes pyruvate oxidation; Increases tubulin binding at the cis side of VDAC	Unknown	Sheldon et al. (2011), Bantug et al. (2018)
Akt/PKB	Prevents closure of VDAC1	Inhibits apoptosis	Unknown	Gottlob et al. (2001)
p38 MAPK	Inhibition of p38 MAPK reduces VDAC1 phosphorylation	Mediates Fas-induced mitochondrial death pathway in CD8^+^ T cells; myocardial apoptosis	Unknown	Farley et al. (2006), Schwertz et al. (2007)
Nek1	Phosphorylates VDAC1	Prevents excessive cell death after injury	Ser193	Chen et al. (2009), Chen et al. (2010)
TLK1/Nek1 axis	Promotes VDAC1 phosphorylation	Prevents apoptosis and maintains mitochondrial integrity	Not specified	Singh et al. (2020)
PINK1	Involved in TOM-VDAC1 complex formation; possibly phosphorylates VDAC	Stabilizes itself and the TOM-VDAC complex; possible role in the regulation of VDAC gating	Not specified	Callegari et al. (2025)

### 1.3 Regulation of VDAC by pH

It is reported that changes in pH gradient affect VDAC function, particularly the channel’s selectivity and conductance (Noskov et al., 2016). A key mechanism for the functional regulation of VDAC through the alteration of pH gradient involves the protonation and deprotonation of amino acids of VDAC protein, particularly acidic residues like aspartate and glutamate (Gincel et al., 2000; Noskov et al., 2016). Experiments on VDACs reconstituted into artificial lipid bilayers have shown that a decrease in pH can reduce channel conductance and alter the composition of ionic current (Noskov et al., 2016). Acidic pH leads to the protonation state of its amino acids, such as the glutamate residue in VDAC1 and VDAC2 (Noskov et al., 2016), that promotes dimerization (Varughese et al., 2021). In addition, lower pH values can lead to changes in ion permeability, increasing anion selectivity and altering overall current-voltage (I-V) relationships (Varughese et al., 2021). While VDAC isoforms share similar structures, their specific responses to pH may vary, e.g., VDAC3 exhibits different characteristics potentially related to its cysteine residues (De Pinto et al., 2016).

### 1.4 Regulation of VDAC by divalent cations

Divalent cations, particularly Ca^2+^, are reported to interact with VDACs, leading to changes in the channel’s conformational flexibility and stability (Ge et al., 2016). The binding of Ca^2+^ can induce VDAC closure, reducing its flexibility and stability in the membrane (Ge et al., 2016). The interaction between VDACs and Ca^2+^ plays a significant role in regulating calcium uptake by mitochondria, which is crucial for many cellular functions (Rosencrans et al., 2021). By modulating Ca^2+^ flux, VDACs contribute to cellular calcium homeostasis, maintaining the right balance of calcium inside and outside the mitochondrion (Rosencrans et al., 2021). Experimental evidence suggests that VDAC1 possesses specific divalent cation-binding sites (Israelson et al., 2007). Trivalent cations like Lanthanum (III) ion (La^3+^) and Terbium ion (Tb^3+^), which bind to Ca^2+^-binding proteins, can induce VDAC1 closure (Shoshan-Barmatz et al., 2015). The three main VDAC isoforms (VDAC1, VDAC2, and VDAC3) exhibit slightly different Ca^2+^ permeability, which is influenced by their specific charge properties (Rosencrans et al., 2021). VDAC1 is more anionic and less Ca^2+^-selective, while VDAC3 is more cationic and more Ca^2+^-selective (Rosencrans et al., 2021).

Magnesium ions (Mg^2+^) have a limited direct role in modulating VDAC, but their influence on mitochondrial calcium levels, a known VDAC regulator, can indirectly affect VDAC’s gating and function (Pilchova et al., 2017).

### 1.5 VDAC & mitochondria-associated membranes (MAM)

**FIGURE 2:**
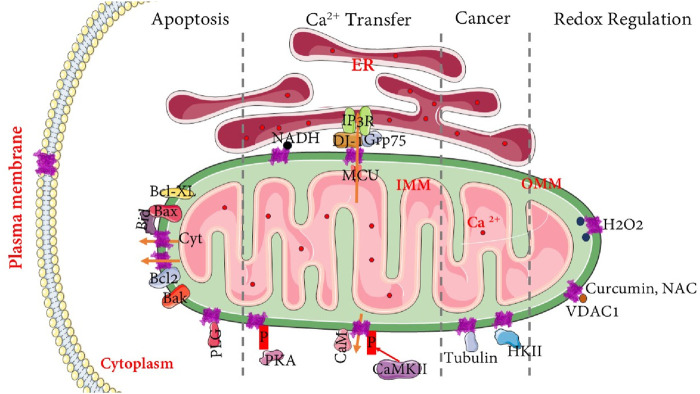
Representative roles of VDAC in apoptosis, cancer, redox regulation, and Ca^2+^ transfer: The schematic diagram depicts VDAC at the outer mitochondrial membrane (OMM) functioning as a central hub. At mitochondria–associated membranes (MAMs), VDAC coordinates ER–mitochondria Ca^2+^ transfer through the IP_3_R–Grp75–MCU complex, while interactions with CaM and CaMKII further modulate Ca^2+^ signaling. During apoptosis, VDAC regulates cytochrome c release via interactions with Bax/Bak, Bcl-2 family proteins, and plasminogen (PLG). In cancer, VDAC supports metabolic reprogramming and cell survival primarily through its association with hexokinase II (HKII) and β-tubulin. In redox regulation, VDAC responds to ROS such as H_2_O_2_, with its activity influenced by antioxidant modulators including curcumin and N-acetylcysteine (NAC). This schematic diagram was adapted and designed with the help of SMART (Servier Medical Art); originally based on SMART imagery. Licensed under CC BY 4.0 (https://smart.servier.com/).

It is reported that Mitochondria-Endoplasmic Reticulum (ER) contact sites, also known as Mitochondria-associated membranes (MAM), tether mitochondria to the ER and regulate a variety of cellular functions, including calcium homeostasis, ROS regulation, and apoptosis (Lee et al., 2019). One of the crucial proteins of MAMs is VDAC, which physically interacts with glucose-regulated protein 75 (GRP75) and inositol triphosphate receptor (IP3R) to regulate ER-mitochondria Ca^2+^ transfer (De Stefani et al., 2012). The VDAC-ER protein interaction is essential for maintaining the structural integrity of MAMs and acts as a key step in ER-mitochondria communication (De Stefani et al., 2012). Under oxidative stress conditions, VDAC’s interaction with the IP3R-Grp75 complex is further stabilized by the Parkinsonism-associated deglycase DJ-1 (also known as PARK7), which supports mitochondrial quality control processes such as mitophagy (Luan et al., 2021). When mitochondria require localized Ca^2+^ uptake to enhance ATP production, closed-state VDAC prioritizes Ca^2+^ transfer over ATP, ADP, and phosphate, which are favored during the open state. This selective permeability helps prevent excessive metabolite leakage that could destabilize mitochondrial membrane potentials. Specifically, the IP3R-Grp75-VDAC complex facilitates mitochondrial Ca^2+^ uptake through the mitochondrial calcium uniporter (MCU) (Barazzuol et al., 2021). These have been explained pictorially in Figure 2. Additionally, fragile X messenger ribonucleoprotein (FMRP) interacts with VDAC to regulate the formation and function of MAMs. Loss of FMRP, the cause of fragile X syndrome, leads to dysregulated ER-mitochondria contact and excessive mitochondrial Ca^2+^ uptake, which impairs synaptic function and cognitive processes. Genetic or pharmacological inhibition of VDAC partially rescues these deficits, highlighting FMRP-VDAC interplay as a critical regulatory axis in mitochondrial and neuronal health (Geng et al., 2023).

## 2 Methods to study VDAC

### 2.1 Experimental methods

Historically, VDAC was initially isolated, purified, and thoroughly examined on phospholipid membranes for its electrophysiological properties (Schein et al., 1976). VDAC was derived from the mitochondrial membranes of *Neurospora crassa* and studied by reconstituting it into liposomes and then patch-clamping it (Wunder and Colombini, 1991). To study human VDAC, two cDNAs corresponding to the transcripts of two separate human VDAC genes were expressed in yeast, and these channels displayed the expected properties of VDAC channels when integrated into planar phospholipid bilayers (Thomas et al., 1991).

The functionality of Voltage-Dependent Anion Channel (VDAC) has been studied to determine its involvement in mitochondrial function, metabolite transport, and cellular signalling. Planar lipid bilayer electrophysiology (BLM) or *in vitro* ion channel reconstitution, protein interactions, mutagenesis, and genetic manipulation are some of the methods used for a better understanding of the VDAC functions.

In our laboratory, mitochondrial Voltage Dependent Anion Channel (VDAC) was isolated from rat/goat brain, liver, and heart as per De Pinto’s protocol (detergents used are Triton-X or Lauryldimethylamine Oxide (LDAO)) (De Pinto et al., 1987; Ben-Hail and Shoshan-Barmatz, 2014; Dearden et al., 2024), later confirmed by Western blotting. The Liquid Chromatography-Mass Spectrometry (LC-MS) showed the ratio of VDAC1, VDAC2, and VDAC3 as 3:2:1 (Malik et al., 2022). VDAC function and its interactions with several proteins and ligands were studied by bilayer electrophysiology, autoradiography (Bera et al., 1995), electrophoresis (Sheldon et al., 2011; Gupta and Ghosh, 2017; Malik et al., 2022), spectrofluorometry (Kerner et al., 2012; Malik et al., 2022), mass spectrometry (Kerner et al., 2012; Malik et al., 2022), molecular docking, etc. (Noskov et al., 2016; Zeth and Zachariae, 2018; Koren and Ghosh, 2022; Koren et al., 2023).

To study the post-translational modifications like phosphorylation and how they regulate the function of VDAC, our group has used *in vitro* methods like phosphorylation studies, in which VDACs were incubated with enzymatic kinases like PKA, and then analyzed through SDS-PAGE, Western blotting, Pro-Q diamond staining (Sheldon et al., 2011; Gupta and Ghosh, 2017; Malik et al., 2022), or autoradiography (Bera et al., 1995). Other methods may include mass spectrophotometry (e.g., LC-MS/MS) for the identification of the phosphorylated residues (Kerner et al., 2012; Malik et al., 2022). VDAC was phosphorylated by several kinases such as PKA, ERK1, JNK3, CaMKII, etc., on the lipid bilayer membrane, and the electrophysiology experiments were performed for the phosphorylated VDAC, and the single/multi-channel data were recorded for further analysis (Bera et al., 1995; Bera and Ghosh, 2001; Gupta and Ghosh, 2015b; 2017; Malik et al., 2022; Koren et al., 2023). Similarly, VDAC was phosphorylated by a specific kinase, say JNK3, in a test tube, and then reconstituted into a lipid bilayer to examine the impact of phosphorylation on its channel activities, such as single-channel conductance and voltage dependency (Gupta and Ghosh, 2015b). The analysis of the above-mentioned experimental data is aided by mathematical and computational modeling, which is discussed below.

### 2.2 Mathematical and computational studies

A theoretical approach based on mathematical and computational modeling can provide comprehensive mechanistic insights that may not be achievable with the existing experimental techniques. One of the critical biophysical properties of ion channels is stochastic fluctuation in current originating from the opening and closing of the channel (Crouzy and Sigworth, 1993). Electrophysiological current recordings from single or multiple ion channels include many forms of noise, which may reflect the diversity in ion flow mechanisms through the channel and along the pore walls. Additionally, noise analysis can be utilized to study the existence of ion channel clusters *in vivo* or *in vitro* biological membranes. The method includes the conversion of time series electrophysiological data into the frequency domain through the application of methods such as the Fourier Transform. An analysis of noise spectra under various experimental conditions can be helpful in the classification of electrophysiological properties of ion channels, such as cooperativity and collective dynamics of ion channels on bilayer lipid membranes (Banerjee and Ghosh, 2005; Shrivastava et al., 2016; Shrivastava and Ghosh, 2021). Similar to the noise analysis of the ion channel current, fractal analysis could be performed to understand the VDAC gating dynamics (Manna et al., 2007). Several other methods, such as molecular dynamics simulations and related computational approaches, can help study VDACs’ interactions with lipids and proteins at the atomic level (Noskov et al., 2016; Cubisino et al., 2024).

Self-organization and collective behavior of ion channels can be analyzed and modeled using statistical mechanical models, such as the Zimm-Bragg model and Ising model (Shrivastava and Ghosh, 2021). These methods include investigating the collective dynamics of ion channel clusters and determining the most probable state (MPS) of these clusters with respect to time, voltage, and other experimental conditions. The method examines the influence of nearest and next-nearest neighbors on the collective dynamics of ion channel clusters to determine positive and negative cooperativity among ion channels within a cluster (Shrivastava and Ghosh, 2021). Self-organization and collective behavior of VDAC could also be investigated through network analysis using various network models, e.g., random network, Hopfield, Ising, small world network, etc. (Hopfield, 1982; Uzun et al., 2017; Gallagher and Carnevale, 2022; Bernáez Timón et al., 2023).

## 3 VDAC-protein interactions

At a cellular level, membrane proteins act as key docking points for the regulation of various signaling pathways. Mitochondrial membrane proteins are key to mitochondrial signaling pathways and the regulation of energy production and distribution. VDAC interacts with various ligands, peptides, and proteins to regulate and bring about the homeostasis of a cell. Some such proteins that have the potential to interact with the purified VDAC include plasminogen, Bax, Bid, Bif, CaM, etc. In this review, as mentioned earlier, a special focus is given to those proteins that regulate mitochondrial physiology. The following are some details of these interactions.

### 3.1 Plasminogen

Recent studies demonstrate that VDAC is a possible ligand for plasminogen (Liang and Bian, 2016). On human endothelial cells, voltage-dependent anion channel 1 (VDAC1) was shown to act as a receptor for plasminogen via Kringle 5 (Li et al., 2014). BLM electrophysiology experiments indicated that plasminogen keeps the VDAC1 channel in a partially closed state, meaning it reduces the conductance of the channel (Banerjee and Ghosh, 2004b). Moreover, Gonzalez-Gronow et al. (2003) demonstrated that VDAC (not isoform specific), expressed on the cell surface of the human neuroblastoma SK-N-SH cell line, has been shown to promote the activation of plasminogen (Pg) through the binding of tissue-type plasminogen activator (t-PA) (Gonzalez-Gronow et al., 2003; 2013). They showed that t-PA binds to human VDAC at a site near its N-terminal region (Gonzalez-Gronow et al., 2003). In addition, they observed that following t-PA-induced Pg activation, VDAC’s NADH-dependent oxidoreductase activity decreased Pg K5, which may be a required mechanism for preventing the cell surface proapoptotic effects of K5. They also examined damaged brain cells, where it was discovered that t-PA-mediated Pg activation was improved by VDAC overexpression (Gonzalez-Gronow et al., 2003). Li et al. (2014) suggested that VDAC, a receptor for plasminogen, can transmit plasminogen-triggered signals that regulate its protein level, subsequently leading to mitochondria-mediated cell apoptosis (Li et al., 2014). They argued that apoptosis occurs through a positive feedback loop, beginning with the initiation of Plasminogen-VDAC1 interaction, followed by activation of protein kinase B, glycogen synthase kinase-3β (GSK3β), enhancing the VDAC1 protein level in endothelial cells (Li et al., 2014). It was demonstrated that plasminogen Kringle 5 (K5) prevented ubiquitin-dependent VDAC1 degradation by phosphorylating VDAC1, most likely at S12 and T107. The AKT agonist, glycogen synthase kinase (GSK) 3β inhibitor, and siRNA all reduced the phosphorylated VDAC1, indicating that K5 enhanced VDAC1 phosphorylation through the AKT-GSK3β pathway. Furthermore, binding between K5 and VDAC1 was observed on the plasma membrane, and K5 helps VDAC1 to translocate to the cell surface. By competitively blocking the association between K5 and cell surface VDAC1, the HKI protein prevented K5 from affecting the AKT-GSK3β pathway (Li et al., 2014).

### 3.2 Bcl-2-associated X protein (Bax)

It has been shown that VDAC2 can regulate Bak via Bax (Chandra et al., 2005), and the mechanism behind this is still unclear. However, one research group believes that VDAC inhibition of Bak activation is dependent on the Bak transmembrane anchor (Lazarou et al., 2010). In mice, Bax and Bak are believed to function as the outer membrane component of the mitochondrial permeability pore in regulating necrotic cell death (Karch et al., 2013). Ma et al. (2014) have shown that mitochondria are targeted by Bax through distinct mechanisms before or during apoptotic cell death (Ma et al., 2014). This mechanism is yet to be unraveled in the scientific literature. To date, it is clear that VDAC2 acts as the mitochondrial platform for Bax retrotranslocation (Lauterwasser et al., 2016). It was reported that VDAC2 is required for Bax to associate with the mitochondrial membrane, and in the absence of VDAC2, Bax was shown to relocalize to other cellular compartments (Lauterwasser et al., 2016). Nucleotides and calcium ions are also involved in Bax retrotranslocation, implying that their transit through VDAC2 may play a significant role (Lauterwasser et al., 2016). Using bilayer electrophysiology, and in the presence of tBid, Bax was reported to increase the pore size of single-channel VDAC (not isoform specific), purified from rat brain (Banerjee and Ghosh, 2004a), leading to a release of apoptogenic molecules, including cytochrome c (Banerjee and Ghosh, 2006). However, the increase in VDAC conductance was reduced significantly upon phosphorylation of VDAC by PKA (Banerjee and Ghosh, 2006). This phenomenon suggests that the increase in pore size due to Bax and tBid interaction is controlled by the action of PKA, which subsequently controls the mechanism of cytochrome c release into the cytosol (Banerjee and Ghosh, 2006). Studies by Chin et al. (2018), using a mouse model at the genetic level, shows that the VDAC2 gene enables the BAX gene to mediate apoptosis and limit tumor development (Chin et al., 2018). They showed that the apoptotic activity of BAX was decreased by genetic deletion of VDAC2, but the interaction of BAX and BAK with mitochondrial complexes containing VDAC1, VDAC2, and VDAC3 was eliminated. Together, their findings demonstrate that VDAC2 is necessary for effective BAX-mediated apoptosis and a clear distinction between the functional effects of BAX and BAK’s interactions with VDAC2 (Ma et al., 2014; Chin et al., 2018). Recently, Champion et al. (2025) reported that VDAC2 is a new therapeutic target that primes myeloma cells for BAK-dependent apoptosis (Champion et al., 2025). However, such experimental evidence is yet to be investigated in order to know the role of other VDAC isoforms, homologs, or paralogs.

### 3.3 Bcl-2 homology 3 (BH3) interacting domain (Bid)

Activation of Bid is brought about by cascades of proapoptotic events. Activated Bid oligomerizes Bak or Bax, leading to the formation of membrane pores that leak cytochrome c (Korsmeyer et al., 2000). It is believed that truncated Bid (tBid) oligomerizes Bak to release cytochrome c (Wei et al., 2000). Cell culture studies highlighted that Bax is colocalized with Bid and VDAC1 (Godlewski et al., 2002). Mitochondrial cristae reorganization and cytochrome c release have been shown as a contribution of Bid protein interaction with lipid cardiolipin at mitochondrial contact sites (Kim et al., 2004). In hippocampal neurons, full-length Bid is sufficient to induce apoptosis (König et al., 2007). Studies by Roy et al. (2009), using mouse embryonic fibroblasts (MEFs) with knockdown of VDAC1, VDAC3, or both knockdown experiments suggest that tBid could play an important role in apoptosis. On the other hand, VDAC2 double knockdown cells, despite adequate tBid-mitochondrial interaction and BAX/BAK expression, lack mitochondrial BAK. Though not as much as double knockdown MEFs, BAK double knockdown MEFs also have decreased tBid sensitivity, which may be due to their high BAX overexpression. Recombinant BAX did, in fact, make VDAC2 double knockdown MEFs more sensitive to tBid. Therefore, by permitting the recruitment of BAK into the mitochondria, VDAC2 plays a critical role in mitochondrial apoptosis, consequently regulating tBid-induced OMM permeabilization and cell death (Roy et al., 2009). Park et al. (2010) reported that *Drosophila* VDAC, particularly the isoform CG6647 (also known as porin), has locomotive defects and male sterility due to the loss of VDAC (porin) (Park et al., 2010). Also, they pointed out that there was an alteration in the mitochondrial morphology and its remodeling processes (Park et al., 2010). At the other end, reactive oxygen species (ROS) signalling triggers a series of Bid-induced mitochondrial membrane permeabilization in the cardiac muscle cell line (H9c2) (Garcia-Perez et al., 2012), where the prominent role of VDAC is indicated, which needs to be uncovered in many respects. As stated earlier, the combined action of tBid and Bax was reported to increase the pore size of VDAC (not isoform specific), purified from rat brain in lipid bilayer membrane (Banerjee and Ghosh, 2004a), which succumbs to the release of apoptogenic molecules, including cytochrome c (Banerjee and Ghosh, 2006). However, the increase in VDAC conductance was reduced significantly upon phosphorylation of VDAC by PKA (Banerjee and Ghosh, 2006). Genomic studies point out that Bid maintains mitochondrial cristae structure (Salisbury-Ruf et al., 2018). Recent studies point out that the interaction between Bid and VDAC1 is an essential factor in determining mitochondrial damage and cell death in neurons (Oppermann et al., 2021). However, direct evidence that confirms their interaction is still lacking in the literature. Genetic and mutational studies would help in understanding the VDAC-Bid protein interaction, in conjunction with other biophysical and biochemical studies. In addition, comparative studies of VDAC isoforms from different sources will enhance a deeper understanding of the role of Bid in VDAC regulation.

### 3.4 Bax-interacting factor-1 (Bif-1)

It has been shown that loss of Endophilin B1/Bif-1 hampers conformational changes undergone by Bax/Bak and thus causes disruption in the mitochondrial apoptotic events (Takahashi et al., 2005). Bif-1 is a multifunctional protein involved in the regulation of apoptosis, mitochondrial morphology, and autophagy, and promotes mitochondrial fragmentation (Etxebarria et al., 2009). Several research groups believe that Bif-1 promotes mitochondrial elongation and survival of neurons (Wang et al., 2014), but no detailed studies have been reported so far. Our experimental findings suggested that Bax and Bif-1 proteins interact with each other and form pores on lipid bilayer membranes (Gupta and Ghosh, 2015a). It was observed that a pore can be formed by an equimolar combination of Bax and Bif-1. The pore conductance ranges between 4.96 and 5.41 nS, including a sub-state with 2.6 nS conductance. However, when monomeric Bax and Bif-1 proteins are examined individually, pore activities are not observed (Gupta and Ghosh, 2015a). On the other hand, VDAC2 has been shown to regulate Bak via Bax (Chandra et al., 2005), and VDAC2 acts as the mitochondrial platform for Bax retrotranslocation (Lauterwasser et al., 2016). During cell stress and apoptotic events, Bif-1 has been shown to interact with Prohibitin-2 and regulate the mitochondrial inner membrane (Cho et al., 2019), suggesting the involvement of VDAC2 and the failure in the electron transport chain; thus, the exchange of energy molecules for cellular activities is highly impacted. These studies suggest that VDACs could be the docking site for these proapoptotic proteins. The conformational changes of Bax/Bak and mitochondria-mediated apoptosis were suppressed with the loss of Bif-1 (Takahashi et al., 2005). This phenomenon indicates that Bif-1 facilitates programmed cell death, potentially via VDAC2 (Chandra et al., 2005; Lauterwasser et al., 2016). The involvement of other VDAC isoforms is still a subject of research and exploration.

### 3.5 Calmodulin (CaM)

Calmodulin (CaM) is a key Ca^2+^-dependent signaling protein abundantly present in the cytoplasm and mitochondria. Several studies suggest that CaM modulates mitochondrial functions by affecting the mitochondrial membrane potential and promoting reactive oxygen species (ROS) production (Odagiri et al., 2009; Kwong and Molkentin, 2015). Although the exact mitochondrial target of CaM remains unclear, VDAC, a highly abundant outer mitochondrial membrane protein involved in these functions, is a possible candidate.

Bilayer electrophysiology experiments demonstrate that CaM binds to VDAC purified from rat brain and regulates its conductance and permeability without changing its voltage dependence properties (Koren et al., 2022). CaM reduces single-channel conductance by approximately 40%–50% and modulates VDAC’s open probability (Koren et al., 2022). Additionally, CaM decreases VDAC’s overall gating charge, lowers Cl^−^ permeability, and favors Ca^2+^ permeability (Koren et al., 2022). The purified VDAC is a mixture of all three isoforms, but LC-MS analysis indicates that VDAC1 is at the highest proportion (Malik et al., 2022), and thus, the contribution to the conductance and gating is believed to be predominantly from VDAC1. However, some reports suggest that in cardiac myocytes, VDAC2 was shown to regulate the resting Ca^2+^ spikes but not action potential-induced Ca^2+^ signaling (Subedi et al., 2011), suggesting an indirect regulation of CaM enzymatic activities. To date, there are no *in vivo* reports on VDAC-CaM interaction as well as isoform-specific interactions.

Fluorescence and bioinformatics analyses reveal a complex, nonlinear interaction between CaM and VDAC, most likely involving multiple CaM-binding sites on VDAC’s outer-loop regions (Koren et al., 2022). These findings suggest that CaM modulates VDAC gating, thereby influencing ion and metabolite transport across the outer mitochondrial membrane. This interaction may play an important role in cellular homeostasis, oxidative stress responses, and metabolic regulation.

### 3.6 Hexokinase (HK)

Hexokinase plays an essential role in mitochondrial energy metabolism. In 1986, Nakashima et al. reported for the first time that 65% of the total hexokinase of the cell can bind to VDAC on the mitochondrial outer membrane (Nakashima et al., 1986). In the brain, hexokinase I, a major isoform, binds to the mitochondrial membrane up to 90% through VDAC1 (Zaid et al., 2005). The binding of HK-I to VDAC1 leads to a reduction in its conductance (Zaid et al., 2005; Abu-Hamad et al., 2008). VDAC1 controls the flow of ATP between mitochondria and the cytosol, making it vital for cellular energy homeostasis (Camara et al., 2017; Varughese et al., 2021). The flux of ATP is regulated by a complex feedback mechanism involving the interaction between hexokinase and VDAC (Pastorino and Hoek, 2008; Perevoshchikova et al., 2010; Roberts and Miyamoto, 2014; Haloi et al., 2021). When VDAC is blocked by hexokinase, ATP cannot pass through the channel, which is necessary for oxidative phosphorylation. This interaction, mediated primarily by the N-terminal domain of hexokinase I, stabilizes its binding to VDAC1 and influences VDAC gating properties. Through this mechanism, cells can effectively switch VDAC on or off to maintain metabolic homeostasis or respond to fluctuating energy demands. Post-translational modifications of VDAC and conformational changes in hexokinase further ensure precise control over mitochondrial metabolite exchange (Sheldon et al., 2012; Lemeshko, 2023). A computational model was developed to thermodynamically quantify the role of VDAC1-hexokinase I (HKI) interaction in regulating the outer mitochondrial membrane potential (OMP) by formulating nonlinear equations and solving them computationally. The analysis suggests that metabolically dependent generation of OMP in brain mitochondria, modulated by factors such as VDAC1-HKI binding, changes in VDAC’s voltage gating, and permeability, could represent a physiological mechanism controlling brain energy metabolism (Lemeshko, 2018). Several reports suggest that even HKI binds and interacts with VDAC2 (Choudhary et al., 2014). It is reported that a decrease in HKI concentration in the spinal cord increases VDAC1’s binding to particular superoxide dismutase 1 (SOD1) variants linked to amyotrophic lateral sclerosis type I, which in turn promotes the formation of toxic SOD1 aggregates, mitochondrial dysfunction, leading to death of motor neurons (Israelson et al., 2010; Magrì et al., 2016). Recent studies demonstrated that HKI binds directly to a charged membrane-buried glutamate in mitochondrial VDAC1 and VDAC2 (Bieker et al., 2025). However, more elaborate studies are still needed to find their possible interaction with VDAC3.

Furthermore, multiple studies have demonstrated that the separation of hexokinase from mitochondria enhances the oligomerization of VDAC, which facilitates NLRP3 (nucleotide-binding domain, leucine-rich family, pyrin domain-containing-3) inflammasome formation and activation (Baik et al., 2023), hence apoptosis. Complex formation between hexokinase II and VDAC1 has also been shown to take place with some additional features in recent years (Haloi et al., 2021; Bieker et al., 2025). *In vivo* studies using human muscle cells and proteomics screening of phosphorylated mitochondrial proteins identified VDAC1 S215 as a phosphorylation site (Zhao et al., 2011). Haloi et al. (2021) report that the phosphorylation site is located at the binding interface of HKII/VDAC1 (Haloi et al., 2021).

### 3.7 Other protein interactions with VDAC

Other proteins that interact with VDAC are also reported by several other groups, some of which are described briefly in this section.

Bcl-2 interacting protein 3 (BNIP3), another proapoptotic protein, has been shown to interact with VDAC (not isoform specific), which induces the mitochondria to release endonuclease G (Zhang et al., 2014), and it is thought that VDAC is involved in this process. Studies by Huang et al. (2014) using the non-small cell lung carcinoma (NSCLC) cell line showed that Myeloid cell leukemia-1 (Mcl-1) binds strongly to VDAC1 and 3 and appears to have a higher affinity for VDAC1 than Bcl-xL (Huang et al., 2014). The interaction of Mcl-1 and VDAC increases lung cancer cell motility through calcium-dependent ROS production (Huang et al., 2014). Impaired functional communication between the L-type calcium channel and mitochondria contributes to metabolic inhibition in the Duchenne muscular dystrophy (mdx) heart (Viola et al., 2014). In yeast, mitochondrial porin (VDAC) has been shown to play pivotal functions in phospholipid metabolism (Miyata et al., 2018). These have been believed to take a dual role: metabolite transport and protein transfer at the outer and inner membrane, respectively (Ellenrieder et al., 2019). TOM22 at the membrane is shown to associate with VDAC1 (Sakaue et al., 2019). Mice upon dietary restriction, and their tissues’ extract co-immunoprecipitation analysis shows the interaction between Apolipoprotein E (ApoE) and VDAC, revealing its important role in mitochondrial function (Rueter et al., 2023; 2024). In cardiac myocytes, they were observed to be colocalized (Chen et al., 2020).

Moreover, the mitochondrial complexome plays a crucial role in metabolic regulation and the import of cytosolic precursor proteins into mitochondria (Sakaue et al., 2019; Wang et al., 2023). The involvement of VDAC is quite predictable from the conclusion made by Zhuang et al. (2024), i.e., mitochondrial volume regulation is mediated by an ion channel that impacts the dynamics in the mitochondrial structure and function (Zhuang et al., 2024). Overall, the regulation of VDAC modulates cell death (Dubey et al., 2016).

Several reports suggest the formation of complexes of VDAC1/2 with creatine kinase (Schlattner et al., 2001) and other cytosolic proteins, including free tubulin and erastin (Rostovtseva et al., 2008; Maldonado et al., 2013), amyloid beta, and phosphorylated tau protein (Manczak and Reddy, 2012). More extensive studies are needed to understand the involvement of various isoforms, homologs, or paralogs from various sources of organisms. Massa et al. (2000) demonstrated that VDAC1/2 mRNA and protein expression are normal in the muscular dystrophy X-linked (mdx) mouse model, but at several developmental stages, VDAC3 mRNA was noticeably downregulated (Massa et al., 2000). They suggest that this discovery raises the possible role of VDAC3 expression in the early pathogenic processes of mdx muscular dystrophy (Massa et al., 2000). In hippocampal neurons, it has been shown that VDAC1/2/3 participates in amyloid beta-induced toxicity and interacts with plasma membrane estrogen receptors (Marin et al., 2007). In addition, various experimental evidences suggest that purified VDAC reconstituted into a lipid bilayer membrane could interact with alpha synuclein (Gurnev et al., 2015; Rostovtseva et al., 2015; Jacobs et al., 2019; Hoogerheide et al., 2021; Rajendran et al., 2022), transactive response DNA binding protein 43 (TDP-43) (Davis et al., 2018), and superoxide dismutase 1 (SOD1) (Israelson et al., 2010). Recent studies suggest that the mitoNEET protein can bind with VDAC and regulate its gating in a redox-dependent manner, and thus interfere with the mitochondrial function (Lipper et al., 2019). In addition, the translocator protein (TSPO) interacts with VDAC and triggers a ROS-mediated inhibition of mitochondrial quality control (Gatliff et al., 2014). Actin (G-actin, but not F-actin) interacts with *Neurospora crassa* VDAC, yeast VDAC (whose isoform is not known), and human VDAC. Actin facilitates the voltage gating but reduces the VDAC conductance (Xu et al., 2001; Roman et al., 2006). Mitochondrial Rho GTPase 1 (MIRO-1) is also reported to interact with *Caenorhabditis elegans* VDAC and regulate the mitochondrial membrane potential. MIRO-1 regulates the VDAC1 activities through its direct interaction, whereas fragmented or damaged mitochondria contain higher levels of MIRO-1 (Ren et al., 2023). This interaction relies on the residues E473 of MIRO-1 and K163 of VDAC1 (Ren et al., 2023). Several other candidate proteins that can regulate VDAC1 are desmin (Li et al., 2016), αβ-crystallin (Diokmetzidou et al., 2016), microtubule-associated proteins (Guzun et al., 2012), etc. For details please see Table 2.

**TABLE 2:** VDAC-protein interaction.

Protein	Effect on VDAC	Plausible consequences	References
Bax	Promotes pore formation via oligomerization of VDAC1	Promotes cytochrome c release and apoptosis	Adachi et al. (2004), Banerjee and Ghosh (2004a)
tBid	Modulates VDAC1 gating; enhances Bax/Bak pore formation	Promotes cytochrome c release and apoptosis	Wei et al. (2000), Godlewski et al. (2002), Banerjee and Ghosh (2004a)
Bak	Activated at the VDAC2 platform, Forms membrane pores with Bax	Promotes cytochrome c release and apoptosis	Chandra et al. (2005), Lauterwasser et al. (2016)
Bif-1	Facilitates Bax/Bak activation at VDAC1-associated sites	Promotes cytochrome c release and apoptosis	Takahashi et al. (2005), Gupta and Ghosh (2015a)
Calmodulin (CaM)	Binds to VDAC in a Ca^2^-dependent manner, alters gating	Perturbs Ca^2+^/ion flux, may sensitize mitochondria to apoptosis (our hypothesis)	Koren et al. (2022)
Bcl-xL	Inhibits VDAC closure	Maintains metabolic flux, suppresses early apoptosis	Arbel et al. (2012)
Plasminogen	Closes VDAC1	Impairs mitochondrial function	Adachi et al. (2004), Banerjee and Ghosh (2004b)
Amyloid-β (Aβ)	Blocks VDAC1 channel conductance; Alters voltage-gating behavior	Mitochondrial dysfunction in Alzheimer’s disease, disrupted metabolite transport, and increased free radical production	Manczak and Reddy (2012)
α-Synuclein	Reversibly and partially blocks VDAC1 conductance, changes ion selectivity from anionic to cationic, and increases Ca^2+^ permeability	Regulation of mitochondrial Ca^2+^ signalling, potential mitochondrial dysfunction in Parkinson’s disease	Rostovtseva et al. (2015)
Tubulin	Causes fast, reversible closures	Acts as a regulator of VDAC permeability to ATP and ADP	Rostovtseva et al. (2008)

## 4 Post-translational modifications (PTMs)

Some of the major post-translational modifications (PTMs) of voltage-dependent anion channels (VDACs) may include phosphorylation, acetylation, O-GlcNAcylation, ubiquitination (Wu et al., 2023), and oxidative modifications, e.g., nitrosylation. These PTMs alter VDAC function by changing channel gating, protein-protein interactions, and stability, playing crucial roles in cellular energy production, metabolism, cell death pathways, and responses to stress.

PTMs can signal VDACs to promote or inhibit programmed cell death (apoptosis) depending on the specific modification and cellular context. Protein-protein interactions are either driven or halted through decisive PTMs that can affect the ability of VDACs to interact with other proteins, influencing various cellular pathways. In addition, changes in PTMs could be a cellular stress response, helping to regulate VDAC activity and maintain cellular homeostasis. PTMs like acetylation, oxidation, succination, etc. have been extensively reviewed by some authors (Kerner et al., 2012; Pittalà et al., 2021).

### 4.1 Phosphorylation of VDAC

As discussed in the introduction, protein phosphorylation plays a significant role in a wide range of cellular processes. It is one of the key post-translational modifications, which in turn regulates biochemical pathways (Ardito et al., 2017; Vitrac et al., 2019; Zhong et al., 2023). Phosphorylation greatly influences the function of transport proteins, e.g., efficiency and the mechanism of ion transport across the membranes (Ismailov and Benos, 1995; Lim et al., 2016; Ardito et al., 2017; Vitrac et al., 2019; Ford et al., 2022; Zhong et al., 2023).

It is well known that modulation of a mitochondrial membrane can be induced by endogenous phosphorylation of membrane proteins such as G-protein coupled receptors (GPCRs) and other transport proteins embedded within the mitochondrial membranes (Ma et al., 2022; Wong et al., 2023; Perri et al., 2024; Pan et al., 2025). As an indirect mechanism, the transport efficiency is regulated in many ways; one way is through phosphorylation, which is empowered by the hydrolysis of adenosine triphosphate (ATP). Different protein kinases, such as protein kinase A (PKA) (Bera et al., 1995; Sheldon et al., 2011), c-Jun N-terminal Kinase-3 (JNK3) (Gupta and Ghosh, 2015b; 2017), extracellular signal-regulated kinase 1 (ERK1) (Malik et al., 2022), Ca^2+^/calmodulin-dependent protein kinase II (CaM kinase II) (Koren et al., 2023), etc., have been reported to phosphorylate VDAC.

In the most commonly studied phosphorylation sites on human voltage-dependent anion channels, e.g., VDAC1, VDAC2, and VDAC3, the specific residues affected, and their functional consequences vary with isoform and context. Phosphorylation of VDAC1 can regulate processes like apoptosis, cardioprotection, and metabolism. Some of the significantly identified sites are mentioned here (Kerner et al., 2012; Ravi et al., 2021). Serine and threonine appear to be the most favorable phosphorylated sites in VDACs. In VDAC1, phosphorylation at S35 is observed to decrease the susceptibility of VDAC1 to oligomerize and form large pores (Mishra et al., 2022; Natarajan et al., 2023). Phosphorylation by the kinase Nek1 at S193 prevents cell death by keeping the VDAC1 channel in a closed state, which prevents the efflux of pro-apoptotic molecules like cytochrome c (Chen et al., 2009; 2010). While phosphorylation of S215 can disrupt the interaction between HKII and VDAC1 (Haloi et al., 2021), T51 phosphorylated VDAC1 by GSK3β has been shown to cause its dissociation from Hexokinase II (HKII), as well as HKII dissociation from the outer mitochondrial membrane (Pastorino et al., 2005; Haloi et al., 2021). In a cardiac model, VDAC1 phosphorylation at T165 was observed to be associated with cardioprotection during ischemia or reperfusion injury. However, a non-phosphorylatable T165A mutation showed cytoprotection, suggesting phosphorylation here may promote cell death (Mishra et al., 2022). Moreover, Y80 and 208 in rat brain VDAC1 (Ballif et al., 2007) and S12, 101, 102, 104, 136, and T107 in HeLa cells VDAC1 (Distler et al., 2006; 2007; Olsen et al., 2006), were found to be phosphorylated.

To date, fewer phosphorylation sites have been functionally characterized on VDAC2 (Kerner et al., 2012). It has been reported that S115 and T118 are the phosphorylated residues in VDAC2, which were experimentally identified in HeLa cell lines, but their functional relevance is not yet defined (Distler et al., 2006; 2007; Olsen et al., 2006). In the rat brain’s VDAC2, T207 (Ballif et al., 2007), and T237 (Olsen et al., 2006) were identified as phosphorylation sites through a mass spectrometry analysis.

Reports from various research groups have identified a few phosphorylation sites on VDAC3, but their number may be rising, while their functional roles are still being explored (Distler et al., 2006; 2007; Olsen et al., 2006). Their reports suggest that T33 and S241 were the phosphorylated residues of VDAC3, purified from rat liver mitochondria and in HeLa cells, that correspond to PKA and PKC consensus sites, respectively (Distler et al., 2006; 2007; Olsen et al., 2006). Also, a phosphorylation at T49 was documented in rat brain (Ballif et al., 2007).

#### 4.1.1 Protein kinase A (PKA)

The mitochondrial VDAC purified from rat brain/mouse liver and reconstituted in bilayer lipid membrane is phosphorylated by the cAMP-dependent protein kinase, also known as protein kinase A (PKA) (Bera et al., 1995; Sheldon et al., 2011). Reported studies on human hepatoma cells HepG2 revealed that VDAC (not isoform specific) permeability for the mitochondrial respiratory substrates is regulated through channel phosphorylation by PKA, increasing the sensitivity to tubulin inhibition (Sheldon et al., 2011). On the other hand, we are yet to identify the specific phosphorylation sites and determine which amino acids in VDAC are involved. Experimental observations on bilayer electrophysiology of VDAC phosphorylated by PKA show that phosphorylation does not affect the current level and the opening probability in the positive clamping potentials but leads to a lowering of current magnitude and opening probability in the negative clamping potentials (Bera and Ghosh, 2001). Open probability patterns analysis indicates that there are two gating modes of VDAC; the negative voltage sensor (gate) undergoes modification due to phosphorylation (Bera and Ghosh, 2001). Furthermore, as stated earlier, the increase in VDAC conductance (an outcome of tBid and Bax interaction) was reduced significantly upon phosphorylation of VDAC by PKA (Banerjee and Ghosh, 2006). This phenomenon suggests that the increase in pore size due to Bax and tBid interaction is controlled by the enzymatic action of PKA, which subsequently controls the mechanism of cytochrome c release into the cytosol (Banerjee and Ghosh, 2006). More *in vivo* studies will reinforce our understanding and confirm the consequent effects of this *kinase*, and perhaps it will open a gateway for therapeutic development. Isoform-specific studies and identification of phosphorylation site(s) are yet to be explored, and mass explorations in other organisms are still due for future research. Given the state of the art, we are unable to comment on the structural and conformational changes, if any, that occur during this phosphorylation event. We believe some amino acid residue displacement might have taken place, but these have yet to be studied on a larger scale.

#### 4.1.2 c-Jun N-terminal kinase 3 (JNK3)

It has been shown that VDAC (purified from rat liver mitochondria, not isoform specific) is phosphorylated by c-Jun N-terminal kinase 3 (JNK3), which is known to be activated during environmental stress, and interferes with the channel’s functioning. Phosphorylation and dephosphorylation are responsible and accountable for the closure and reopening of the channel (Gupta and Ghosh, 2015b; 2017). In addition, a massive reduction in the opening probability and a modification in the voltage dependence had been observed (Gupta and Ghosh, 2015b; 2017). However, understanding the exact mechanism required more detailed studies. These drastic changes might have serious physiological consequences, like JNK3-dependent mitochondria-mediated apoptosis. *In vivo* experiments and isoform-specific studies will greatly enhance our understanding of the role of VDAC regulation in mitochondria or even in other organelles that potentially express VDAC proteins. Studies on structural and conformational changes can be studied using imaging techniques like microscopy and structural techniques like cryo-EM would be useful tools to go deep into the interaction of JNK3 and VDAC.

#### 4.1.3 Extracellular signal-regulated kinase (ERK)

Activation of extracellular signal-regulated kinase-1/2 (ERK1/2) has been shown to occur in cells with tumor progression (Yang et al., 2008), cell proliferation (Sun et al., 2015), stress response (Sánchez et al., 1994), and metastasis (Lu et al., 2012). Our findings suggest that VDAC (purified from goat brain and not isoform specific) is phosphorylated by ERK1, leading to the reduction of its single-channel conductance (Malik et al., 2022). The loss in the conductance was partially recovered by alkaline phosphatase, indicating it was partly reversible. The mass spectrometric analysis suggests Threonine 33, Threonine 55, and Serine 35 to be the phosphorylated sites (Malik et al., 2022). In HeLa cells, ERK1 is shown to be physically associated with VDAC1, interfering with the transport mechanism involving signaling and metabolism (Galli et al., 2009). In mitochondria, steroidogenic acute regulatory protein localization is regulated through mitochondrial fusion and ERK activities (Duarte et al., 2014), suggesting the involvement of VDAC. It would be interesting to see if ERK2 has the potential to phosphorylate all the VDAC isoforms.

#### 4.1.4 Calmodulin-dependent protein kinase II (CaMKII)

In the mitochondria of smooth muscle cells, CaMKII has been shown to control mitochondrial mobility, migration, and neointima formation (Nguyen et al., 2018). Kostic et al. (2014) reported that voltage-gated Ca^2+^ channels are regulated by Ca^2+^/calmodulin-dependent protein kinase II in resting sensory neurons (Kostic et al., 2014). ROS-mediated CaMKII activation is observed to contribute to abnormalities in calcium handling and impaired muscle contraction in Barth Syndrome (Liu et al., 2021). Calmodulin kinase, a calcium-dependent protein kinase, has been shown to phosphorylate VDAC (Koren et al., 2023). The CaMKII drastically reduces the channel current and holds the channel in the open state, referring to a loss in its voltage sensitivity (Koren et al., 2023). It is important to note here that CaM kinase has multiple isoforms, and thus, it is believed that all CaM isoforms may not interact with VDAC. Also, it is relevant to the fact that VDAC isoforms may interact with CaM kinases in different modes of action. These puzzling permutations and combinations of interactions are an intriguing area for future research. Isoform-specific or organelle-specific or even organism-specific studies will add vast knowledge to the literature.

#### 4.1.5 Other protein kinases phosphorylating VDAC

Baines et al. (2003) showed through transgenic mice and coimmunoprecipitation studies that protein kinase C can phosphorylate VDAC1 (Baines et al., 2003), but electrophysiology and other studies are yet to be done. Several research groups reported that GSK-3β (Sheldon et al., 2011), Akt/PKB (Gottlob et al., 2001), and p38 mitogen-activated protein kinase (MAPK) inhibition significantly reduced the phosphorylation of VDAC1, which is a known mitochondrial regulator (Schwertz et al., 2007). In CD8^+^ T cells, p38 MAPK has been shown to mediate the Fas-induced mitochondrial death pathway (Farley et al., 2006), where the involvement of VDAC is expected. Studies using human CD8^+^ T cells showed that the GSK3β inhibition enables binding of HK-I to VDAC1, promoting pyruvate oxidation (Bantug et al., 2018). VDAC1 interacts directly with never-in-mitosis A-related kinase 1 (Nek1), which phosphorylates VDAC1 at S193 to prevent excessive cell death after injury (Chen et al., 2009; 2010). Recent studies show that apoptosis and mitochondrial integrity can be prevented through VDAC1 phosphorylation under the influence of the tousled-like kinase 1 (TLK1) (Singh et al., 2020). Phosphatase and tensin homolog (PTEN)-induced kinase 1 (PINK1) is involved in the stabilization of itself and the formation of the TOM-VDAC complex at the outer mitochondrial membrane (Callegari et al., 2025). So, there is a possibility that PINK1 might interact with VDAC. Notably, isoform-specificity of VDAC complex formation with TOM has not been studied in detail.

Vast numbers of enzymes and kinases are present in the cellular environment, and thus, there is always a possibility that they might interact with VDACs at some phases of the organism’s developmental stage. This aspect is yet to be explored in combination with other molecular and genetic studies, both *in vitro* and *in vivo*. Numerous physiological changes, such as alterations in the signaling pathway, are highly likely to occur, which may result in either gain of functions or loss of functions. Localization or trafficking of VDACs and/or their interacting partners is still poorly understood. Moreover, isoform-specific and organism-specific studies remain largely unexplored.

### 4.2 Acetylation

Acetylation is a known post-translational modification at specific lysine residues in VDAC1, VDAC2, and VDAC3, particularly on the N-terminus (Kerner et al., 2012). The specific numbering of lysine residues can vary slightly depending on the protein source (e.g., human vs. rat) and the method used. However, the overall location within the protein structure and functional roles remain consistent. In VDAC1, VDAC2, and VDAC3, lysine residues are located on both the outer surface of the β-barrel structure and within the central pore (Ahmad et al., 2020). Their specific placement and modifications are important for function and regulation, including metabolite transport and apoptosis. The precise location of each lysine differs by isoform, with VDAC1 and VDAC2 being extensively studied than VDAC3. In VDAC1, K197 (Lysine-197), K200, K201, and K224 are part of a cluster facing the inner channel, interacting with the N-terminal α-helix (Kerner et al., 2012). The N-terminal region of VDAC2 is longer by 11 residues than VDAC1, which possesses extra interaction sites for protein/ligand binding inside the channel’s pore. It has been reported that mouse liver VDAC1 is acetylated at positions K41, K122, and K132 (Schwer et al., 2009). In the mouse liver and heart, another site of VDAC1 acetylation was found at K237 (Yang et al., 2011). The acetylation sites in VDAC2 of mouse liver mitochondria that have been identified are K32, K75, and K121 (Kerner et al., 2012). The acetylation sites in VDAC3, which can impact protein function, are K20, K61, K63, K109, K226 (in mouse liver mitochondria), and K28 (human liver mitochondria) (Kerner et al., 2012). Moreover, the gating mechanism of VDAC3 involves hydrolysis of disulfide bonds that were formed between its N-terminal region and the posterior portion of the pore. Remarkably, VDAC1 deacetylation has been shown as a mechanism of inducing an antiapoptotic process in cardiomyocytes by some authors (Tong et al., 2017). However, the functional effect of acetylation of VDAC isoforms is yet to be determined.

### 4.3 O-GlcNAcylation

It is reported that VDAC can undergo O-GlcNAcylation, but the specific target site remains unknown (Jones et al., 2008). These experiments were carried out in an *in vivo* mouse model and cardiac myocytes. Jones et al. (2008) reported from their *in vivo* mouse model studies that ischemic preconditioning increases O-GlcNAc levels, sufficient to reduce infarct size following *in vivo* myocardial ischemia/reperfusion injury (Jones et al., 2008). On the other hand, cardiac myocytes experience a decrease in O-GlcNAc levels during lethal cell injury. The reduction in O-GlcNAc levels during early cardiomyocyte damage was observed to coincide with the loss of mitochondrial membrane potential, and thus increasing O-GlcNAc levels slows the decline in the loss of mitochondrial membrane potential and cell death (Jones et al., 2008). Isoform-specific studies would enhance our understanding of therapeutic development to counter pathological conditions. Nevertheless, exploring various homologs and paralogs of VDACs would enlarge the applications towards mitochondrial physiology and pathophysiology.

### 4.4 Ubiquitination

Wu et al. (2023) reported recently that ubiquitination of VDAC1 on specific sites restricts its oligomerization and mitochondrial DNA release in liver fibrosis (Wu et al., 2023). They found that site-specific ubiquitination of VDAC1 at lysine 53 by Parkin, a ubiquitin ligase, interrupted VDAC1 oligomerization and prevented mtDNA release into the cytoplasm under stress. The ubiquitination-defective VDAC1 K53R mutant predominantly formed oligomers that resisted suppression by Parkin. Hepatocytes expressing VDAC1 K53R exhibited mtDNA release and thus activated the STING signaling pathway in hepatic stellate cells, and this effect could not be abolished by Parkin. Furthermore, they suggest that Parkin prevents liver fibrosis by ubiquitinating VDAC1 at a particular location, which halts VDAC1 oligomerization and prevents mtDNA release (Wu et al., 2023). It has been reported that VDAC1 and VDAC3 are ubiquitinated, but not VDAC2 (Sun et al., 2012). Reports suggest that the VDAC3 ubiquitination is directly proportional to the duration of hypothermia conditions (Zhao et al., 2020).

### 4.5 Oxidation

In VDAC, the oxidative modifications primarily occur on cysteine residues exposed to the mitochondrial intermembrane space, a region where ROS are abundant (De Pinto et al., 2016). The cysteine and methionine residues within the three mammalian VDAC isoforms (VDAC1, VDAC2, and VDAC3) can undergo various levels of oxidation (De Pinto et al., 2016). Human VDAC1 has two cysteine residues that are targets for oxidation (De Pinto et al., 2016). First, Cys127 faces the lipid bilayer and is less likely to undergo reversible oxidation (De Pinto et al., 2016). Second, Cys232 faces the hydrophilic pore of the channel and is exposed to the intermembrane space (IMS), making it a favorable candidate for oxidation by reactive oxygen species (De Pinto et al., 2016). Human VDAC2 has nine cysteines, significantly more than VDAC1 (De Pinto et al., 2016). Many of these are positioned in loops that protrude into the IMS, making them highly susceptible to oxidation, e.g., Cys36, Cys65, Cys199, and Cys216. In mouse VDAC2, these residues were found to have significant changes in their oxidation status under stress conditions. Human VDAC3 has six cysteines, several of which are exposed to the IMS and can exist in various oxidation states (De Pinto et al., 2016). Mass spectrometry and mutagenesis studies show that these specific cysteines (Cys2, Cys8, and Cys122) can influence the protein’s stability and channel activity depending on their redox state. Cysteines 2 and 8 are located in the N-terminal domain of VDAC3 and can form a disulfide bridge (De Pinto et al., 2016). VDAC3, along with other isoforms, have cysteines that can be overoxidized to sulfonic acid (De Pinto et al., 2016). Cysteine has the potential to undergo reversible oxidative modifications, as well as deformation of disulfide bonds (-S-S-) and irreversible oxidation to sulfinic acid (-SO_2_H) and sulfonic acid (-SO_3_H) in VDACs (De Pinto et al., 2016). This overoxidation has been observed specifically in VDACs and not in other mitochondrial membrane proteins (De Pinto et al., 2016). In addition to cysteine, methionine residues can also be oxidized to methionine sulfoxide and methionine sulfone (Saletti et al., 2018). Several research groups have proposed that oxidation of cysteine in VDAC provides a protective mechanism to the cells (Saletti et al., 2018).

### 4.6 S-Nitrosylation

S-Nitrosylation is a type of oxidative modification and a post-translational modification where a nitrosyl group (-NO) is added to the sulfur atom of a cysteine residue in a protein, forming an S-nitrosothiol bond (Chang et al., 2014). This process is a form of nitric oxide (NO)-mediated signaling and is reversible (Martínez-Ruiz and Lamas, 2004). The three human VDAC isoforms (VDAC1, VDAC2, VDAC3) can all be S-nitrosylated (Das and Steenbergen, 2012; Okazaki et al., 2015). The S-nitrosylation of VDAC3 is vividly significant due to its ability to functionally activate VDAC3, and this precise activation is dependent on the redox state of its cysteine residues (Okazaki et al., 2015). For example, S-nitrosylation of VDAC3 may be essential for sperm motility, an inevitable physiological process in the reproductive system (Sampson et al., 2001). Noteworthy, S-nitrosylation of VDAC, along with other mitochondrial proteins, can impact mitochondrial function and is implicated in conditions like ischemic injury and autism spectrum disorder (ASD) (Das and Steenbergen, 2012; Chang et al., 2014).

### 4.7 S-Glutathionylation

S-Glutathionylation of the mitochondrial VDAC is a reversible post-translational modification where a sulfur atom of a cysteine residue in VDAC forms a disulfide bond with glutathione (GSH) (Chan et al., 2018). Researchers have developed methods like GluICAT (Glutathione-modified Isotope-Coded Affinity Tag) to quantify S-glutathionylation in proteins like VDAC by differentially tagging glutathionylated and native cysteines (Chan et al., 2018). S-glutathionylation has been observed in various cellular processes, with mitochondrial proteins being a major focus due to the mitochondrion’s central role in redox homeostasis and cell death pathways (Xiong et al., 2011; Vrettou and Wirth, 2022). Hitherto, data and information about S-glutathionylation of various isoforms of VDACs are not yet available in the scientific literature.

## 5 Interaction of VDAC with small molecules/ligands

In this section, we discuss the role of various ligands involved in mitochondrial physiology and the role of VDAC. It is a well-known fact that many antioxidants, drugs, and phytochemicals, being hydrophobic in nature, are able to cross the cell membrane and reach mitochondria. It is highly possible that they bind to the mitochondrial surface via VDAC before they are transported across the mitochondrial membrane, and thus interfere with the VDAC channel conductance. During pathophysiological conditions, the steady-state equilibrium of the cells is disturbed, which results in an alteration in the proportion of ions and metabolites distribution between mitochondria and cytosol and triggers changes in the metabolic pathways. Ultimately, such cells tend to generate more ROS, which in turn could affect VDAC conductance. The antioxidants, drugs, and phytochemicals may help prevent and subside the aforesaid ion and metabolite imbalance. Some of the commonly used compounds that have the potential to interact with VDAC are homocysteine, quinidine, thymoquinone, curcumin, etc., whose effects have been discussed below.

### 5.1 Homocysteine (HCY)

Methionine is an essential amino acid that can naturally metabolize to various cellular components, including HCY (Martínez et al., 2017; Sanderson et al., 2019). There is evidence that excess intracellular HCY accumulation occurs, which makes the cardiovascular system vulnerable to various pathological conditions (Steed and Tyagi, 2011; Ganguly and Alam, 2015). The accumulated HCY can transform into its thiol form, known as homocysteine thiolactone, which is more reactive than the HCY itself. The excess HCY thiolactone has the potential to interfere with the mitochondrial membrane, and the mitochondrial proteins are thought to act as its docking site. Moreover, it is reported that HCY enhanced ERK1/2 protein expression levels and oxidative stress induced cytochrome c translocation and mitochondrial dysfunction and cardiac dysfunction in rats (Gu et al., 2010; Wang et al., 2020). Our group has shown that the thiol form of HCY binds to VDAC on bilayer lipid membrane and increases its conductance (Koren and Ghosh, 2022). However, under induced oxidative stress, its conductance was partially recovered from the effects caused due to HCY-thiolactone interactions (Koren and Ghosh, 2022). The interaction between VDAC1 and HCY (free and thiol form) was confirmed with molecular docking and fluorescence studies (Koren and Ghosh, 2022). The estimated binding energy for Homocysteine-thiolactone to VDAC is −4.7 kcal mol^−1^ (Koren and Ghosh, 2022). Hitherto, there is no evidence of modulation of VDAC2 and VDAC3 by HCY-thiolactone. Hence, it is very difficult to compare the binding affinity of VDAC1 with other isoforms. Homocysteine can directly inhibit the activity of various complexes within the electron transport chain, leading to decreased ATP (energy) production (Austin et al., 1998). In addition, homocysteine increases the production of reactive oxygen species (ROS) through its auto-oxidation or by activating oxidant systems, which directly damages mitochondrial components (Austin et al., 1998).

### 5.2 Quinidine

Quinidine is an antiarrhythmic drug known to treat irregular heartbeat (Wenckebach, 1923; Rakza et al., 2025). The action of quinidine is due to inhibition of the entry of Na^+^ ions, thereby causing changes in the flux of K^+^ ions (Klein et al., 1960; Choi et al., 1972; Imaizumi and Giles, 1987). Also, various types of K^+^ channels are inhibited upon the application of quinidine (Yatani et al., 1993; Nenov et al., 1998; Bednarczyk et al., 2004; Tamargo et al., 2004; Koepple et al., 2017). Moreover, it affects the transport of Ca^2+^ across the mitochondrial membrane (Harrow and Dhalla, 1976), oxidative phosphorylation, and other mitochondrial respiration pathways (Batra, 1976; Komai and Berkoff, 1979). It is reported that quinidine decreases the one-way flow of potassium and magnesium ions in mitochondria of rats’ liver (Diwan, 1986). Thus, a large number of ion channels in the cell and mitochondrial membrane are the targets of the antiarrhythmic drug quinidine. VDAC1, being one of the major mitochondrial ion channels, could be a potential target for quinidine as well. Our group has shown that quinidine binds to VDAC and modulates its channel conductance on BLM (Malik and Ghosh, 2020b). *In silico* molecular docking studies suggest that quinidine interacts with VDAC1 at the glutamic acid residue, specifically Glu-206 (Malik and Ghosh, 2020b). As per our knowledge, there is no direct evidence showing a significant affinity of quinidine for VDAC2 or VDAC3.

### 5.3 Hydrogen Peroxide (H_2_O_2_)

Hydrogen peroxide (H_2_O_2_) is one of the key molecules that is involved in the neuropeptide secretion and various cell signalling processes (Lennicke et al., 2015; Di Marzo et al., 2018; Sies and Jones, 2020; Jia and Sieburth, 2021). Indeed, mitochondria act as the major source and generator of reactive oxygen species (ROS) (Edmondson, 2014). During pathophysiological conditions, the concentration of H_2_O_2_ and other ROS increases abnormally (Sies, 2014). This leads to the creation of oxidative stress, which favors activation of various death signalling pathways (Loot et al., 2009). In the absence of superoxide dismutase (an enzyme that protects cells from oxidative damage), mitochondrial proteins, e.g., VDAC protein from heart mitochondria of adult male Wistar rats (Han et al., 2003), become the target for H_2_O_2_-mediated oxidation (Ugarte et al., 2010). Han et al. (2003). Furthermore, it was demonstrated that VDAC can regulate the movement of ROS, especially superoxide anions, from mitochondria to cytosol (Han et al., 2003). These reports suggest that hydrogen peroxide might be a strong candidate that can interact with VDAC and regulate its function. Our experimental results of BLM electrophysiology indicate that H_2_O_2_ interacts with VDAC and modulates its channel gating (Malik and Ghosh, 2020a; Koren and Ghosh, 2022; Siddiqui et al., 2023). In fact, our report suggests that H_2_O_2_ has the potential to increase the VDAC single-channel conductance in lipid bilayer membrane through lipid peroxidation (Malik and Ghosh, 2020a). Unlike VDAC1, VDAC3 contains six cysteine residues with five clustered in the intermembrane space and one in the pore’s interior (Reina et al., 2022). Under conditions of high oxidative stress, these cysteine residues might get overoxidized. VDAC3’s affinity with oxidative stress is characterized by the overoxidation of its cysteine residues in an oxidizing environment rich in reactive oxygen species (ROS) (Reina et al., 2022). This cysteine overoxidation indicates that VDAC3 can serve as a marker for the oxidative load within mitochondria and may function as a component of the ROS signaling pathway (Reina et al., 2016a). VDAC3 is therefore implicated in cellular responses to oxidative stress and mitochondrial dysfunction (Reina et al., 2016a). VDAC2’s specific affinity with H_2_O_2_ is not broadly documented, while reports cover studies primarily on the role of VDAC isoforms in general or other specific isoforms like VDAC1 and VDAC3’s roles in oxidative stress. However, research on lamprey VDAC2 (Lr-VDAC2) shows that it suppresses H_2_O_2_-induced apoptosis by inhibiting pro-apoptotic proteins like BAK (Zhang et al., 2024). On the whole, VDAC proteins in general are oxidized by ROS, with H_2_O_2_ attributed to increasing VDAC single-channel conductance.

High concentrations of H_2_O_2_ rapidly and significantly disrupt cellular energy metabolism by damaging mitochondria, which in turn affects ATP transport in rat heart myocytes (Tatsumi and Kako, 1993). In addition, H_2_O_2_ inhibits the mitochondrial ATP synthase enzyme complex, which is crucial for producing ATP. The adenine nucleotide translocator (ANT), which transports ATP out of the mitochondria, is highly susceptible to H_2_O_2_ and is a primary target of oxidative damage (Tatsumi and Kako, 1993; Lemasters, 2021). At moderate concentrations, H_2_O_2_ triggers programmed cell death (apoptosis) via the intrinsic mitochondrial pathway (Saito et al., 2006). It promotes the release of cytochrome c from mitochondria, which activates caspase-9 and the downstream “executioner” caspase-3, leading to cell death (Saito et al., 2006). At higher concentrations, H_2_O_2_ depletes ATP stores so severely that the energy-dependent process of apoptosis is blocked. This instead triggers necrosis, which is characterized by cell swelling and rupture (Saito et al., 2006).

### 5.4 N-acetyl L-cysteine (NAC)

NAC is one of the commonly used antioxidants for cellular studies (Elbini Dhouib et al., 2016; Tenório et al., 2021), which reduces free radical species in the cells (Lu et al., 2009; Aldini et al., 2018; Ezeriņa et al., 2018; Zhitkovich, 2019; Pedre et al., 2021). This indicates its potential use as a therapeutic drug (Elbini Dhouib et al., 2016). NAC acts as a mitochondrial protector by reducing ROS production. NAC helps maintain normal mitochondrial function and prevents oxidative stress-induced damage (Zheng et al., 2024). Moreover, NAC can prevent apoptosis by stabilizing mitochondrial membranes and inhibiting the release of pro-apoptotic factors, which are often triggered by oxidative stress (Zheng et al., 2024). NAC does not directly facilitate ATP transport in cells of Wistar rats. However, by protecting mitochondrial function and oxidative phosphorylation, it indirectly contributes to the production and maintenance of adequate ATP levels (Anna et al., 2021). It is reported and suggested that NAC scavenges ROS and increases cellular glutathione levels, both of which help protect mitochondria from oxidative stress (Halasi et al., 2013). Bilayer electrophysiology experiments show that NAC binds to VDAC and modulates its activity, particularly in the context of oxidative stress (Siddiqui et al., 2023). As per *in silico* studies, binding of NAC to VDAC takes place through specific amino acids like lysine and glutamic acid (Siddiqui et al., 2023). This interaction protects VDAC1 from the effects of H_2_O_2_ (Siddiqui et al., 2023). As per our knowledge, there is no evidence suggesting a direct affinity or interaction between VDAC2/VDAC3 and N-acetylcysteine (NAC), pending further studies.

### 5.5 Sodium azide (NaN_3_)

Several toxicological studies have revealed that NaN_3_ is one of the hazardous compounds, especially when inhaled (Klein-Schwartz et al., 1989; Weiss, 1996; Tat et al., 2021). Nonetheless, it is still widely used as a food preservative, agricultural pest control, and in hospital and laboratory settings as a disinfectant. In cells, this compound is known to disrupt mitochondrial respirations (Palmieri and Klingenberg, 1967) via cytochrome-c-mediated blocking of the electron transport chain (Bennett et al., 1996; Leary et al., 2002), ultimately activating caspase-dependent apoptotic pathways (Zuo et al., 2019; Amer et al., 2024). Moreover, Zuo et al. (2019) reported an increase in the production of ROS on application of NaN_3_ to PC12 cells (Zuo et al., 2019). They observed a decreased ATP production and loss of mitochondrial potential in PC12 cells (Zuo et al., 2019). Sodium azide inhibits cytochrome oxidase, a key enzyme in the mitochondrial electron transport chain, which leads to a rapid and severe depletion of cellular ATP levels (Bowler et al., 2006). The reduction in intracellular ATP results in activation of K_ATP_ channels by sodium azide, leading to cell hyperpolarization (Harvey et al., 1999). This altered membrane potential can then influence the state of other voltage-gated channels (Harvey et al., 1999). VDACs, being one of the major components of the outer membrane of mitochondria, the above-mentioned studies strongly indicate VDACs as the potential target for NaN_3_. Preliminary investigations by our group show that NaN_3_ interacts with VDAC.

### 5.6 Curcumin

Curcumin is an active compound extracted from the plant *Curcuma longa* (Kocaadam and Şanlier, 2017), highly useful in health and medicine, pharmacy, and kitchens in Indian households (Hatcher et al., 2008). The antioxidant properties of this compound have made it one of the favorites for cellular studies, especially when oxidative stress is in operation (Ak and Gülçin, 2008). Curcumin inhibits the mitochondrial ATP synthase, leading to reduced ATP production and decreased oxygen consumption, causing a fatal energetic impairment in tumor cells (Bianchi et al., 2018). It demonstrates anti-apoptotic properties by reducing the expression of apoptosis-related genes like Bcl-2 and Bax (Jiang et al., 2024). It enhances the action of several enzymes, such as superoxide dismutase and catalase, and thus decreases the free radical generation in cells (Nelson et al., 2006; Barzegar and Moosavi-Movahedi, 2011). In cortical neurons, it has been shown that curcumin protects mitochondria from oxidative damage and attenuates apoptosis (Zhu et al., 2004). A series of experimental reports suggests that VDAC1 may be involved in the above-mentioned cellular events and processes (Shoshan-Barmatz et al., 2020; Rosencrans et al., 2021). Tewari et al. (2015) have shown that curcumin directly interacts with expressed human VDAC1 at the α-helical N-terminus on the bilayer lipid membrane (Tewari et al., 2015). Specific residues involved in the N-terminus are suggested to be Lys15, Arg18, and Asp19, along with Tyr198 in the inner channel wall (Tewari et al., 2015). Following these, our group has extended and studied the effect of H_2_O_2_ on the interaction between rat brain VDAC and the role of curcumin (Malik and Ghosh, 2020a). It was observed that H_2_O_2_ has the potential to increase the VDAC single-channel conductance on the lipid bilayer membrane, and curcumin can partially reverse the effects (Malik and Ghosh, 2020a). While direct studies on curcumin’s affinity to VDAC2 may be limited, existing data provide some insights into its potential interactions. hVDAC2 is known for its role as an anti-apoptotic protein and features an additional 11-residue N-terminal extension (NTE) compared to other VDAC isoforms. This NTE, along with VDAC2’s higher cysteine content, may influence its interaction with molecules like curcumin, which can be affected by both protein structure and oxidative states. Experimental studies and accumulation of evidence on VDAC3-Curcumin direct interaction/binding is an intriguing future scope of research.

### 5.7 Thymoquinone (TQ)

TQ is a phytochemical extracted from the seeds of a plant commonly called black cumin (*Nigella sativa*) (Gholamnezhad et al., 2016). It is known for its anti-inflammatory (Chehl et al., 2009), antioxidant (Badary et al., 2003), anti-apoptotic (Kelso et al., 2001), and anti-cancer effects (Gali-Muhtasib et al., 2008). Chemically, it is lipophilic in nature and can cross cell membrane barriers easily (Salmani et al., 2014; Darakhshan et al., 2015; Goyal et al., 2017; Ceylan et al., 2023; Imam et al., 2024). In fibroblast cells, TQ and its cationic derivatives have been demonstrated to reduce the effect of mitochondrial H_2_O_2_ and its production; as a consequence, H_2_O_2_-induced apoptosis was alleviated (Severina et al., 2013). Moreover, it is reported that TQ can act as a neuroprotective agent (Severina et al., 2013). In contrast, another report suggests that TQ can induce and increase ROS production, which leads to the activation of apoptotic pathways in chondrocytes (Isaev et al., 2020). In tumor cell lines, TQ can cease cell proliferation through an unknown mechanism involving mitochondrial dysfunction and thus cell death (Yu and Kim, 2013). Despite the above-mentioned contrasting views and findings, we understand the importance of TQ in mitochondria-mediated cell death. Current research does not provide a direct or specific mechanism describing the interaction between thymoquinone (TQ) and the voltage-dependent anion channel (VDAC). However, evidence suggests TQ indirectly influences VDAC and its function by modulating the wider apoptotic signaling pathway, particularly involving the Bcl-2 family of proteins. Preliminary experiments show that TQ interacts with goat brain VDAC. On the whole, TQ has been shown to act as either anti-apoptotic or pro-apoptotic, depending on the cellular conditions. In cancer cells, such as melanoma, TQ can induce apoptosis by increasing ROS levels and hindering the Jak2/STAT3 signaling pathway (Raut et al., 2021). More experiments that target VDAC as a binding partner would help researchers gain more insights about the mechanism of TQ action and other associated reactions.

### 5.8 Regulation of VDAC with HgCl_2_

HgCl_2_ causes a significant reduction in the mean single-channel conductance of Rat VDAC (Malik and Ghosh, 2020c). Upon HgCl_2_ treatment, VDAC conductance decreases substantially (Malik and Ghosh, 2020c). The gating charge of VDAC, which is crucial for its voltage-dependent opening and closing, is also affected (Malik and Ghosh, 2020c). HgCl_2_ treatment leads to a significant change in the gating charge, indicating an alteration in the electrical properties that govern channel gating (Malik and Ghosh, 2020c). While VDAC1 is generally anion-selective in its open state, the presence of HgCl_2_ could influence its ion selectivity due to a change in the gating charge (Araragi et al., 2003; Malik and Ghosh, 2020c). In asymmetrical HgCl_2_ concentrations, a change in the cation-to-anion permeability ratio has been observed (Malik and Ghosh, 2020c). While direct, explicit research detailing the precise molecular interaction of HgCl_2_ with VDAC to trigger apoptosis is not extensively highlighted, the general mechanisms of VDAC1-mediated apoptosis strongly suggest its involvement. Given that HgCl_2_ induces cytochrome c release and affects mitochondrial permeability transition, and VDAC1 is a critical player in both these processes, it is highly probable that HgCl_2_ exerts its apoptotic effects, at least in part, by modulating VDAC function. In a bilayer electrophysiology experiment, the interaction of VDAC with HgCl_2_ leads to a dysfunctional channel characterized by decreased ion conductance, altered voltage-gating, and significant changes in ion selectivity, likely due to mercury binding to critical sulfhydryl groups within VDAC (Malik and Ghosh, 2020c). This phenomenon provides insights into the molecular mechanisms of mercury toxicity at the cellular level, particularly concerning mitochondrial function. Exposure to HgCl_2_ has been shown to interact with and disrupt the function of the Voltage-Dependent Anion Channel (VDAC), a crucial outer mitochondrial membrane protein (Araragi et al., 2003).

### 5.9 Interaction of VDAC with NADH and other metabolites

It is well established that VDAC acts as a primary gatekeeper and regulates the passage of various ions, metabolites, and molecules across the mitochondrial outer membrane to meet the cellular needs (Shoshan-Barmatz and Gincel, 2003). VDAC is known for its permeability to both NADH and NAD+, allowing their dynamic equilibrium to exist across mitochondria (Baghel and Thakur, 2019; Yang S. et al., 2024; Yang et al., 2024 Y.). The flux of NADH and NAD+ through VDAC is very important as it maintains cellular redox potential balance and supports metabolic processes like glycolysis in the cytosol and oxidative phosphorylation within the mitochondria (Varughese et al., 2021; Yang Y. et al., 2024; Yang et al., 2024 S.). It is reported that the overall NAD+/NADH ratio across the mitochondrial membrane is critical for cellular energy balance (Cantó et al., 2015; Xie et al., 2020; Yusri et al., 2025). Thus, NADH interaction with VDAC is highly significant. Recent evidence suggests that NADH binds to a specific pocket on the inner surface of VDAC1 (Heslop et al., 2022; Conti Nibali et al., 2024) and regulates its gating (Zizi et al., 1994). The binding of NADH leads to a reduction in conductance of hVDAC1 mainly by steric hindrance that occludes the pore, thereby promoting a closed state of this channel (Rostovtseva et al., 2002).

Gincel et al. (2000) reported that VDAC1 has a specific binding site for glutamate and modulates the channel gating behavior and its conductance (Gincel et al., 2000; Gincel and Shoshan-Barmatz, 2004). In addition, they highlighted that acetylcholine and dopamine are permeable to VDAC1, which strongly suggests that they might have a specific binding site (Gincel et al., 2000; Gincel and Shoshan-Barmatz, 2004). Other water-soluble metabolites and ions regulated by VDAC include ATP, ADP, nucleotides, citrate, etc. (Rostovtseva and Colombini, 1996; Hodge and Colombini, 1997; Budelier et al., 2017). Previous reports suggest that transport of NADH across mitochondrial membranes in yeast is possible through VDAC1 and VDAC2 but not by VDAC3 (Xu et al., 1999).

However, there are a large number of compounds reported to interact with VDAC. It is beyond the scope of this manuscript to describe each compound interacting with VDAC. Hence, we have enlisted the above-mentioned compounds in a tabular form (Tables 3, 4) for the convenience of the readers.

**TABLE 3:** Physiological modulator of VDAC.

Agent	Effect on VDAC properties	Plausible consequences	Reference(s)
Homocysteine (HCY)	Binds (thiol form) to VDAC, increases channel conductance	Promotes oxidative stress, mitochondrial dysfunction, and cardiac dysfunction	Wang et al. (2020), Koren and Ghosh (2022)
Hydrogen peroxide (H_2_O_2_)	Binds to VDAC, modulates channel gating, and increases conductance via lipid peroxidation	Induces oxidative stress on VDAC	Malik and Ghosh (2020a), 2020b
N-Acetyl L-Cysteine (NAC)	Interacts with VDAC, modulates channel conductance	Reduces oxidative stress caused by H_2_O_2_ on VDAC	Siddiqui et al. (2023)
NADH	Binds to specific pocket of VDAC1, reduces conductance (steric hindrance)	Modulates mitochondrial metabolism	Rostovtseva et al. (2002), Heslop et al. (2022)
Glutamate, Acetylcholine, Dopamine	Bind to VDAC, modulate gating and conductance	Mitochondrial swelling and cytochrome c release	Gincel et al. (2000), Gincel and Shoshan-Barmatz (2004)
Nucleic Acids (tRNA in plants)	VDAC-34 and VDAC-36 show differential binding to tRNA	Facilitates tRNA transport across the mitochondrial membrane (in plants)	Salinas et al. (2006), Salinas et al. (2014)
Superoxide	A reactive oxygen species (ROS) that can cause VDAC-dependent permeabilization of the outer mitochondrial membrane, leading to the release of cytochrome c	ROS-induced cell death	Yapryntseva et al. (2024)
ATP	High concentrations of ATP can bind to a specific pocket on VDAC promoting a closed state and reducing the flux of metabolites	Regulation of cellular energy production	Heslop et al. (2022)
Cytosolic proteins (Tubulin, *α*-synuclein)	The polyanionic C-terminal domains of these proteins can physically block the VDAC channel at lower transmembrane potentials by blocking the pore	Disruption of mitochondrial membrane potential and dysregulation	Rostovtseva et al. (2021)

**TABLE 4:** Pharmacological modulator of VDAC.

Name of compound	Activator/Blocker	Mechanism and effect	Potential use	References
Erastin	Activator	Promotes ferroptosis (a type of cell death) by acting on mitochondrial VDAC and accelerating oxidation. It is selective for tumor cells with oncogenic RAS.	Cancer therapy	DeHart et al. (2018)
SW016789	Activator	Induces VDAC1-targeted insulin hypersecretion and calcium influx in beta cells. Causes a reversible, non-apoptotic endoplasmic reticulum (ER) stress response	Type 2 diabetes research	Israelson et al. (2005)
VBIT compounds (VBIT-3, VBIT-4, VBIT-12)	Blocker	Inhibit VDAC1 oligomerization, preventing the formation of larger, pro-apoptotic pores. VBIT-4 also reduces mitochondrial DNA release	Treating apoptosis-associated disorders, such as neurodegenerative and cardiovascular diseases. Lupus research	Kim et al. (2019), Belosludtsev et al. (2024)
DIDS sodium salt	Blocker	Inhibits both the anion transport activity of VDAC1 and VDAC1 oligomerization. It also inhibits caspase activation	Cancer research and studies involving anion exchange inhibition	Ben-Hail et al. (2016)
NSC 15364	Blocker	Directly interacts with VDAC1 to prevent its oligomerization, which is associated with inhibiting apoptosis	Apoptosis research	Lv et al. (2024)
WEHI-9625	Blocker	A tricyclic sulfone that binds to VDAC2, enhancing its ability to inhibit apoptosis. It is effective against mouse BAK but not human BAK or BAX.	Understanding VDAC2-mediated apoptosis inhibition	van Delft et al. (2019)
Quinidine	Blocker	Binds to VDAC, modulates channel conductance	Prevents cell proliferation and induces apoptosis in some systems	Ru et al., (2015)
Sodium azide (NaN_3_)	Activator	Potential interaction with VDAC (our hypothesis)	Induces mitochondria-mediated apoptosis	Zuo et al. (2019)
Curcumin	Blocker	Directly interacts with VDAC1, modulates conductance, reverses H_2_O_2_ effects	It may protect mitochondria from oxidative damage by its antioxidant properties	Tewari et al. (2015), Malik and Ghosh (2020a)
VA molecules (VDAC Antagonists)	Blocker (Antagonist)	Binds to VDAC1 with higher affinity than NADH	Promotes Cell death in cancerous cells through the mitochondria-mediated metabolic pathway	Conti Nibali et al. (2024)

## 6 VDAC-nucleic acid interaction

There are some reports that demonstrate the involvement of plant VDACs in the transport of tRNA (Transfer-RNA) across the mitochondrial outer membrane (Salinas et al., 2014). While VDAC is very well-known for its ability to transport metabolites, studies have shown that VDAC can also play a crucial role in tRNA translocation (Salinas et al., 2006; 2014). However, VDAC is not the sole tRNA receptor on the outer membrane; other proteins like the translocase of the outer membrane (TOM), e.g., TOM20 and TOM40 from the TOM complex, are also involved in tRNA binding and import (Salinas et al., 2006). Plant VDACs, i.e., VDAC-34 and VDAC-36, exhibit differential interaction with tRNA. VDAC-34 has a stronger binding affinity to tRNA than VDAC-36 (Salinas et al., 2014). This difference in binding is not limited to tRNA but extends to other nucleic acids as well, suggesting a potential specialization in their functions (Salinas et al., 2014). This phenomenon is limited to plant cells and has not been reported in animal cells to date, which suggests an evolutionary divergence of plant cells. It may be mentioned here that in the present review, we are confined to animal cells & tissues. Thus, detailed discussions on plant VDACs are not included in the present review.

## 7 Role of lipids in regulation of VDAC

It is widely known that lipids are extremely essential in cellular physiology, including membrane transport, osmosis, homeostasis, etc. Phosphatidylcholine is a major constituent of cell membranes and associated organelles (Kanno et al., 2007). However, the ratio of other lipids varies in inner cell organelles (Jackson et al., 2016; Decker and Funai, 2024). In mitochondria, the percentage of lipids in the outer mitochondrial membrane is as follows: Phosphatidylcholine (PC) is ∼50% of OMM lipids, phosphatidylethanolamine (PE) is ∼30% of the OMM, and cardiolipin (CL) is ∼20% (De Kroon et al., 1997; Ingólfsson et al., 2014; Jackson et al., 2016; Funai et al., 2020; Lafargue et al., 2025). Recent reports suggest that lipids are tightly bound to the transporter proteins and ion channels (Stieger et al., 2021), and thus, careful consideration and the choice of lipids are extremely important. The types of lipid or a mixture of lipids utilized would consequently affect *in vitro* experimental results (Stieger et al., 2021). Phosphatidylcholine remains a good choice of lipid for bilayer electrophysiology experiments with VDAC, as PC mimics bilayer membranes well (Johnson et al., 1973). Notably, our group has explored the possibility of cardiolipin to make an artificial bilayer membrane to reconstitute rat/goat brain VDAC for our bilayer electrophysiology experiments (Malik and Ghosh, 2020c). It was observed that VDAC conductance (4.3 ± 0.18 nS), the current vs. applied potential (IV) plot, and other electrophysiological characteristics remained unchanged (Malik and Ghosh, 2020c). It was also observed that divalent cations such as Hg^2+^ bind well with CL (Malik and Ghosh, 2020c). Betaneli et al. (2012) showed that the nature of lipids determines hVDAC1 assemblies on BLM (Betaneli et al., 2012). Phosphatidylglycerol present in BLM in higher concentration tends to cause hVDAC1 oligomerization (Betaneli et al., 2012). Interestingly, during apoptosis, the ratio of phosphatidylglycerol to other lipids has been shown to significantly increase (Betaneli et al., 2012; Wupperfeld et al., 2022; Lafargue et al., 2025), which could be due to the aforesaid hVDAC1 oligomerization. On the other hand, some reports suggest that CL tends to disrupt the hVDAC1 oligomerization on the BLM; hence, it should combat apoptosis (Betaneli et al., 2012). In addition, several reports have indicated that cholesterol and other sterols can modulate VDAC gating, and VDAC can affect the distribution of cholesterol in the OMM (Campbell and Chan, 2007; Mlayeh et al., 2010).

## 8 VDAC current noise & fractal analyses

Generally, noise is defined as an irregular fluctuation in the signal whose origin is obscure (Bak et al., 1987). As per the recent understanding, noise analysis can uncover many hidden properties of the system (Crouzy and Sigworth, 1993; Banerjee and Ghosh, 2005; Banerjee et al., 2006). Following the noise analysis method described in a previous section, we have demonstrated that the VDAC open single-channel current noise displays power law behavior, precisely a 1f pattern (Banerjee and Ghosh, 2005; Banerjee et al., 2006), known as pink noise. It indicates that the dynamics of a single VDAC follow autocorrelation in the time series. However, the pattern changes to white noise, 1f0 (noise representing random or non-correlated noise) pattern on the interaction of VDAC with other proteins, ligands, and phosphorylation. This could be a simple yet promising biophysical method to identify VDAC interactions with other molecules and chemical groups (Banerjee et al., 2006). In general, the origin of noise in ion channel current is related to ion transport and the fluctuation of their conformations (Shrivastava et al., 2016). We are in agreement with the idea that the above-mentioned observations on VDAC single-channel dynamics are due to the existence of a phenomenon called self-organized criticality (SOC) (Bak et al., 1987; Banerjee and Ghosh, 2005). Given the state of the art, there is a lot of controversy regarding the conceptual interpretation of the power-law noise (Siwy and Fuliński, 2002). Subsequent analysis of the VDAC gating (opening and closing) noise on BLM shows a 1f pattern. The general consequence of a system showing 1f noise is the existence of a self-similar pattern. In order to investigate this, fractal analysis was carried out on the VDAC gating time series data, and some interesting features, like fractal dimension, were deciphered (Manna et al., 2007).

## 9 VDAC oligomers & clusters

VDAC exists as assemblies or groups on the native mitochondrial membrane. Biophysical investigations of VDAC revealed the presence of an optimal number of channels that form clusters exhibiting collective gating behavior beyond the physical or spatial size of VDAC oligomers (Shrivastava and Ghosh, 2021). This collective gating behavior may arise out of interactions between neighboring or even non-neighboring channels, either directly or indirectly (Shrivastava and Ghosh, 2021). We define VDAC clusters as those exhibiting collective gating behavior resulting from both direct and indirect interactions among neighbouring channels. The former are referred to as VDAC oligomers.

### 9.1 VDAC oligomers

Since the early 1980s, electron-microscopic investigations of native VDAC in isolated membrane vesicles have shown the supramolecular assembly of VDAC (Mannella et al., 1983). Here, the VDAC channels were observed in hexameric arrangements with two-fold symmetry or as dimeric VDAC pores forming chain-like superstructures (Mannella et al., 1983). Additionally, atomic force microscopy (AFM) images of VDAC channels revealed the existence of various oligomeric forms, such as monomers, dimers, trimers, hexamers, and clusters of up to 20 VDACs (Gonçalves et al., 2007; Keinan et al., 2013).

The process of VDAC1 oligomerization increases with an increase in intracellular calcium concentration. Calcium facilitates the process either by activating an unknown signaling pathway or by increasing VDAC1 expression, leading to apoptosis even in the absence of apoptotic stimuli (Keinan et al., 2013). Also, the oligomeric form of VDAC1 is suggested to play a role in Ca^2+^ transport, which is crucial for mitochondrial calcium homeostasis and signaling (Weisthal et al., 2014; Rosencrans et al., 2021; Sander et al., 2021). The entry of Ca^2+^ into mitochondrial intermembrane space controls the energy metabolism by regulating crucial enzymes of the pathway (Finkel et al., 2015). VDAC oligomerization has been proposed as a molecular signal associated with cytochrome c release during mitochondria-mediated apoptosis (Zalk et al., 2005). A recent report by Takeda et al. (2025) on the cryo-EM resolved structure of the yeast VDAC (represented as Por1) hexamer and mutational studies reveals that oligomeric VDAC not only transports metabolites and ions but also facilitates mitochondrial protein import by binding to unassembled Tom22, a subunit of the Translocase of the Outer Mitochondrial Membrane (TOM) complex. Interestingly, it has been recently reported that protein E (E-2) from SARS-CoV-2 forms a voltage-dependent channel with preference towards monovalent cations similar to VDAC and self-assembles into a homo-pentamer (Cubisino et al., 2024). In addition, VDAC oligomerization helps regulate mitochondrial DNA retention and loss in cooperation with nucleases (Takeda et al., 2025). These findings highlight the functional importance of VDAC oligomerization and were further supported by biophysical investigations into how VDAC oligomerization facilitates collective and cooperative phenomena.

### 9.2 VDAC collective behavior

As the VDAC molecules are present in cluster form, they interact with each other. The membrane lipid composition plays a crucial role in VDAC organization in the mitochondrial outer membrane (Lafargue et al., 2025). This interaction would lead to the collective opening of a certain number of ion channels, depending on the range of interactions. In our earlier reports, it was pointed out that the VDAC cluster organization facilitates collective phenomena (Shrivastava and Ghosh, 2021). The quantification of the size of the cooperative cluster was done by calculating the mean fraction of channels in the open state. In bilayer electrophysiology experiments, after the immediate application of voltage, all the channels present on the membrane become active and show opening, but gradually the channels acquire an optimized state, which is lower than the maximum number of channels present in the bilayer membrane, as indicated by the decrease in current amplitude. It has been observed that approximately 4-5 channels remain open after the cluster system reaches a stable state (Shrivastava and Ghosh, 2021). These interactions may occur via direct physical contact or indirectly through local ionic gradients and diffusion processes (Shrivastava and Ghosh, 2021). Such interactions can ultimately modify the gating behavior of clustered ion channels compared to that of isolated single channels (Kim et al., 2019). Previous reports suggest that cluster organization can alter single-channel conductance and noise characteristics (Banerjee et al., 2006). Therefore, the clustering of VDAC channels may induce significant changes in both the structural (Kim et al., 2019) and functional properties of individual channels (Shrivastava and Ghosh, 2021).

Moreover, modulation in single and one-channel (conductance of one VDAC channel in a cluster) conductance of VDAC and its noise profile due to the presence of neighboring ion channels could be an indication of a collective phenomenon (Malik and Ghosh, 2013). Remarkably, our earlier reports have demonstrated that channel-channel interaction can regulate noise profile and be used as an indicator of functional coupling between the channels (Shrivastava et al., 2016).

## 10 Discussion

In this paper, we have reviewed the modulation of Voltage Dependent Anion Channel (VDAC), mostly from the mitochondria of animal tissues, by various factors, e.g., post-translational modifications like phosphorylation, acetylation, sulphonation, etc., protein & enzyme interactions, ligand/small molecule interactions, nucleic acid interactions, membrane lipid composition, etc., both *in vitro* and *in vivo,* covering work of our group as well as others. The focus of this review article has been on the ion channel electrophysiology experiments. The observations are summarized below.

1. Phosphorylation of VDAC by a variety of kinases leads to closure of the channel.
2. Proteins (apoptotic & anti-apoptotic) tend to partially or fully block the channel, except for a few, e.g., Bax and Bid.
3. Proteins causing neurodegeneration, e.g., amyloid-β, α-synuclein, are reported to have interactions with VDAC1.
4. Oxidative stress caused by various ligands & agents leads to blockade of the channel.
5. There are some ligands, both inorganic & phytochemicals, which can combat the effect mentioned in 4.
6. Clustering (including oiligomerization) of VDACs on the bilayer membrane leads to enlargement of the channel pore, leading to leakage of the channel.
7. Lipid composition of the membrane plays an important role in the regulation of VDAC.
8. Current noise analysis is a powerful method to understand the dynamics of VDAC.

Although most of the above-mentioned inferences are based on bilayer electrophysiology experiments, i.e., *in vitro*, it is reasonable to think that these facts would hold true *in vivo*. If it is so, then these have significant consequences in cellular physiology. For example, closure of the channel pore would inhibit the passage of ATP and other metabolites across the outer mitochondrial membrane. This can lead to (a) dysfunction of the cytosolic metabolism and (b) swelling of the mitochondria, followed by bursting. The obvious results are apoptosis or necrosis. On the other hand, enlargement of the VDAC pore would lead to leakage of metabolites and some unwanted proteins from mitochondria to cytosol, resulting in cell death. While cell death, e.g., apoptosis, is essential for living tissues, e.g., development & differentiation, and combating cell proliferation, it can cause damage to a living system, e.g., neurodegeneration. These have been supported by specific examples as demonstrated below. It may be mentioned here that, keeping in mind the dominant biological importance of phosphorylation of VDAC, the latter has been the focus of our discussion on post-translational modification.

### 10.1 Phosphorylation of VDAC

Table 1 summarizes the effect of VDAC by various kinases and their consequences in living systems. As described in the previous section, a large number of kinases, e.g., PKA, PKC, JNK3 & other MAP kinases, ERK, CaM kinase, etc., have been experimentally proven to modulate VDAC through phosphorylation, mostly by partially or fully blocking the channel, as evidenced by the reduction of channel conductance and open probability. This would mean that the passage of metabolites like ATP from the mitochondrial intermembrane space to the cytosol is hindered. This is expected to lead to energy deprivation in the cytosol, hence slowing down or annihilating metabolic activities. This would lead to cell death, which could be alarming for normal cells. On the contrary, inducing this activity would combat cell proliferation in cancer cells, hence could be a mechanism to resist cancer.

However, for hexokinase, binding and unbinding with VDAC is the natural mechanism of controlling ATP flux and switching on and off the glycolytic cycle through a negative feedback mechanism (Pastorino and Hoek, 2008; Perevoshchikova et al., 2010; Roberts and Miyamoto, 2014; Haloi et al., 2021). This has been discussed in the previous section.

As an exception, when Bax & Bid enlarge the VDAC pore on the membrane (as described in an earlier section in this review), leading to leakage of cytochrome c from the intermembrane space of mitochondria to the cytosol, giving rise to triggering the apoptotic pathway, PKA has been shown to undo this VDAC pore leakage *in vitro*. Thus, PKA should be able to combat apoptosis if the story holds true in cellular systems, and VDAC would play a key role in this phenomenon. We believe this could be a plausible mechanism by which phenomena like neurodegeneration can be resisted in principle.

### 10.2 VDAC-protein interactions

VDAC-protein interaction influences the mitochondrial membrane permeability efficiency (Tomasello et al., 2009; Camara et al., 2017). The signalling pathways involving proapoptotic (Bcl2) proteins are heavily affected (Tsujimoto and Shimizu, 2000; Morris et al., 2021; Yuan et al., 2021; Vogler et al., 2025). This disruption in the mitochondrial membrane potential and energy production is directly or indirectly linked to VDAC and its phosphorylated state (Rostovtseva et al., 2008; Sheldon et al., 2011; Maldonado et al., 2013; Varughese et al., 2021). Table 4 summarizes the effects of various proteins affecting VDAC and the plausible consequences at the cellular level. VDAC interaction with various proteins, in some cases, is dependent on the phosphorylation of the partner proteins, which is phosphorylated by the secondary enzymes or kinases, ultimately regulating the status of VDAC phosphorylation (Lewandrowski et al., 2008; Pastorino and Hoek, 2008; Pittalà et al., 2021). Mutated protein and off-target phosphorylation may result in the formation of aggregated proteins, which may enhance VDAC oligomerization or even membrane disruption (Kim et al., 2019; Haloi et al., 2021; Zong et al., 2024). The oligomerized VDAC may leak apoptogenic agents like cytochrome c and even imbalance the metabolite transport ratio, affecting the translocation of ATP and ADP across the mitochondrial membrane, which again impairs the mitochondrial energy production via glycolysis and the electron transport chain (Shoshan-Barmatz et al., 2015; Heslop et al., 2021; Khaliulin et al., 2024).

### 10.3 Oxidative and other stresses on VDAC

As described in the previous section, certain small molecules, e.g., H_2_O_2_, cause oxidative stress leading to structural changes in VDAC. This would obviously modulate the channel, resulting in inhibiting metabolite transport, e.g., ATP, across the mitochondrial outer membrane, hence cell death. On the other hand, there is a possibility of oligomerization of VDAC due to the action of oxidizing agents (Keinan et al., 2010; Lemeshko, 2023; Wu et al., 2023; Khaliulin et al., 2024), giving rise to leakage of cyt. c and some metabolites to the cytosol and initiation of apoptosis. Interestingly, certain small molecules, e.g., thymoquinone, curcumin, and NAC, are capable of reversing the oxidative effect, thus protecting VDAC as well as the cell. Reina et al. (2016a) suggested that VDAC3, but not VDAC1 or VDAC2, could be a putative sensor of mitochondrial ROS levels (Reina et al., 2016a; 2016b). Until then, VDAC1 had been considered a major player in ROS-induced apoptosis (Brahimi-Horn et al., 2015) and responsible for the translocation of superoxide anion from the inner mitochondrial space (IMS) to the cytosol (Han et al., 2003). However, their recent studies have demonstrated that the expression of VDAC3 protein on the mitochondrial surface protects mitochondria from dreadful consequences like oxidative stress (Reina et al., 2022). They reported that there was a significant increment in the cytotoxic level of redox cyclers like paraquat and menadione, and electron transport chain’s complex I inhibitors such as rotenone, which was shown to promote accumulation of uncontrolled free radicals in the mitochondria (Reina et al., 2022). Transient transfection of the wild type and the cysteine-null mutant VDAC3 protein in HAP1-ΔVDAC3 expressing cells, and their assessment through respirometry, confirmed that VDAC3 cysteines are crucial for the protein’s ability to restrain ROS-induced oxidative stress (Reina et al., 2022).

An elevated level of HCY leads to a synchronous increase in ROS in the isolated mitochondria (Bukharaeva et al., 2015; Wang et al., 2020). Increased concentration of HCY is associated with the apoptosis of adult cardiomyocytes (Sipkens et al., 2007; 2013). Notably, HCY reduced the mitochondrial potential of cardiomyocytes (Sipkens et al., 2007). The exact mechanism of HCY-induced mitochondrial signalling is still illusive, but given that HCY reduces conductance of VDAC, which in turn is a key protein in several apoptotic pathways, there is a chance that HCY-induced disturbance of mitochondrial potential would occur through VDAC. Secondly, HCY is likely to cause oligomerization of VDAC through ROS production, as mentioned in the previous paragraph, leading to leakage of cyt. c, etc., hence apoptosis (Keinan et al., 2010).

As reported earlier, glutamate induces VDAC closure and mitochondrial stress, contributing to excitotoxic apoptosis, especially in neurons. On the other hand, glutamate can induce the oligomerization of VDAC1, which has been linked to increased oxidative stress and cell death (Gincel et al., 2000; Gincel and Shoshan-Barmatz, 2004). This process can be inhibited by certain VDAC inhibitors, like DIDS, which can improve cell survival (Ben-Hail and Shoshan-Barmatz, 2016; Nagakannan et al., 2019). High cytosolic NADH closes VDAC, disrupting energy metabolism and sensitizing cells to apoptosis. Studies have shown that VDAC can be a target for oxidative stress induced by sodium azide, hence oligomerization of VDAC and leakage of apoptogenic molecules. Calmodulin binds to VDAC in a calcium-dependent manner, altering its conductance and gating, which can disrupt mitochondrial ion and metabolite exchange, perturb Ca^2+^ homeostasis, and promote mitochondrial-mediated apoptosis (Israelson et al., 2005). As reported earlier, mercury in various forms interacts with voltage-dependent anion channels (VDACs) (Malik and Ghosh, 2020c), and we believe this would also hold in the mitochondrial outer membrane of cells. As VDACs are crucial for regulating mitochondrial function, their interaction with mercury can disrupt this, leading to the release of molecules that trigger apoptosis.

### 10.4 Role of VDAC in the therapeutic action of phytochemicals & drugs

As discussed earlier, quinidine interacts with VDAC in a way that can partially block it. This could give rise to mitochondrial swelling and cell death. Although there is very little information on the role of quinidine in apoptotic cell death, there is evidence that it prevents cell proliferation and induces apoptosis in human glioma cell lines (Ru et al., 2015). Given the circumstances, there is a good possibility that the therapeutic action of quinidine involves its interaction with VDAC.

Studies show that NAC can interact with VDAC, reducing its conductance and potentially protecting it from oxidative stress caused by H_2_O_2_ (Siddiqui et al., 2023). The protective effect of NAC on VDAC may be due to its antioxidant properties, helping to mitigate oxidative damage to the channel.

Curcumin reduces the conductance of VDAC potentially by stabilizing a closed conformation of the channel, which should logically lead to mitochondrial swelling and cell death (Tewari et al., 2015; Malik and Ghosh, 2020a). Whether curcumin’s therapeutic action is due to apoptosis or not is yet to be established and demands more experimental investigation. Nevertheless, VDAC plays a significant role in the remedial activity of curcumin.

As it is argued that VDAC is involved in the whole process of mitochondria-mediated apoptosis, its interaction with TQ could be the actual mode of therapeutic action of the latter. This suggests a potential role of VDAC in the above-mentioned therapeutic role of thymoquinone, pending further research in order to fully elucidate the specific mechanisms involved.

Table 2 summarizes the effects of various ligands (small molecules) affecting VDAC and the plausible consequences at the cellular level.

### 10.5 Relevance of membrane lipid composition in VDAC function

As mentioned in the previous section, during apoptosis, there’s often an increase in Phosphatidylglycerol (PG) and a decrease in Cardiolipin (CL) levels within the mitochondria. Increased PG levels in the OMM during apoptosis may promote VDAC oligomerization, potentially facilitating the release of apoptotic factors (Rostovtseva et al., 2006; Tang et al., 2024). Decreased CL levels in the OMM during apoptosis can disrupt VDAC oligomerization (Rostovtseva et al., 2006; Tang et al., 2024). As it has been reported that VDAC conductance is very much dependent on the lipid composition of the membrane, VDAC’s role in apoptosis is closely intertwined with the lipid composition of the mitochondrial outer membrane, and alterations in this lipid composition can significantly impact VDAC’s function and its contribution to cell death.

### 10.6 Future prospects of VDAC research

From the present review, it is very clear that extensive experimental research, right from structural to functional, has been carried out on VDAC both *in vitro* and *in vivo*. This has happened because VDAC, which was initially thought to be just a passage for metabolites like ATP on the outer membrane of the mitochondrion, proved to play very important roles in apoptosis, necrosis, switching on and off in the glycolytic cycle, oxidative stress, inter-organelle communications, etc., and their subsequent physiological consequences. Despite the fact that there exists a huge database about VDAC, there is enough scope for future research on VDAC. For example, a lot can be studied regarding the post-translational modifications of VDAC and their biological significance. Investigation of the interactions of VDAC with newer proteins and small molecules (natural, phytochemicals, and synthetic) would open new windows in VDAC research. On the other hand, VDAC has been designated to have evolutionary significance, e.g., prokaryotic (bacterial) porin evolved as mitochondrial porin. Keeping this in mind, it is important to study VDAC isomers from different living objects and investigate their structural and functional homology as well as diversity. Lastly, VDAC’s existence in the plasma membrane raises several questions. Does pl-VDAC play any role as a receptor, in cell-to-cell communication, or cell signaling? Addressing these problems would lead to brilliant research in the field of VDAC.

## 11 Conclusion

In summary, voltage-dependent anion channels (VDACs), major outer mitochondrial membrane proteins, play a significant role in mitochondrial physiology and maintenance of cellular homeostasis. Recent research has gathered evidence on the existence of VDACs in the plasma membrane and other organelles. VDACs are found in plants, animals, bacteria, protists, etc., and exist in various isoforms. In animals, particularly mammals, VDACs are reported to exist in three isoforms, i.e., VDAC1, VDAC2, and VDAC3. To date, the structure of VDAC1 and VDAC2 has been resolved through X-ray crystallography and NMR spectroscopy, but the VDAC3 structure remains unresolved. Like other proteins, VDACs can undergo various forms of post-translational modifications such as phosphorylation, acetylation, O-GlcNAcylation, ubiquitination, and various types of oxidative modifications to modulate and regulate their own functions and other physiological activities. It may be noted that lipid peroxidation and the composition of membrane lipid greatly influence the structure and functions of VDACs, both *in vivo* and *in vitro*. Moreover, VDACs can interact with cytosolic and mitochondrial proteins, including pro-apoptotic and anti-apoptotic proteins, thereby regulating mitochondrial functions such as mitochondrial signaling pathways, mitochondrial membrane potential, and apoptosis, etc. Furthermore, VDACs could interact with various metabolites like NADH and ATP to regulate the production of cellular energy and their needs for growth and survival. Various ligands and other small molecules have the potential to interact with VDACs, wherein they can modulate and regulate VDACs’ functions in several modes of action(s). It is vital to note that VDAC can be regulated by changes in mitochondrial or cellular pH, different divalent cations, and many other factors. Precisely, different isoforms of VDACs may have different sensitivity and responses to interacting proteins, ligands, and metabolites depending on the sources, localization, and even the conformational state of VDAC proteins. The structural and functional implications of VDACs include regulation of Ca^2+^ transfer, apoptosis, development of cancerous cells, and redox regulation (to name a few of them). In physiological conditions, VDACs can organize themselves and function either as oligomers or clusters, showing collective behavior to execute mitochondrial and cellular functions.

On the whole, we may conclude that phosphorylation, protein interaction, oxidative stress, and ligand interactions with VDACs in some way or other have the potential to cause apoptosis of cells, provided the nature of the above-mentioned effects is valid in living tissues. This could have dual effects. For example, the aforesaid apoptosis can combat cell proliferation and cancer. On the other hand, in some tissues, say neuronal cells, the VDAC-mediated cell death can lead to disease, e.g., neurodegeneration. Thus, in addition to the regulation of the metabolic (ATP) flux between the mitochondria and the cytosol, the importance of VDAC in living systems lies in its role in cellular apoptosis, whether it be cancer therapy or neurodegeneration.
